# Epidemiological and Virological Characteristics of Influenza Viruses Circulating in Cambodia from 2009 to 2011

**DOI:** 10.1371/journal.pone.0110713

**Published:** 2014-10-23

**Authors:** Srey Viseth Horm, Sek Mardy, Sareth Rith, Sovann Ly, Seng Heng, Sirenda Vong, Paul Kitsutani, Vannra Ieng, Arnaud Tarantola, Sowath Ly, Borann Sar, Nora Chea, Buth Sokhal, Ian Barr, Anne Kelso, Paul F. Horwood, Ans Timmermans, Aeron Hurt, Chanthap Lon, David Saunders, Sam An Ung, Nima Asgari, Maria Concepcion Roces, Sok Touch, Naomi Komadina, Philippe Buchy

**Affiliations:** 1 Institut Pasteur du Cambodge, Réseau International des Instituts Pasteur, Phnom Penh, Cambodia; 2 World Health Organization, Phnom Penh, Cambodia; 3 Communicable Disease Control Department, Ministry of Health, Phnom Penh, Cambodia; 4 Influenza Division, National Center for Immunization and Respiratory Disease, Center for Disease Control and Prevention, Atlanta, Georgia, United States of America; 5 Centers for Disease Control and Prevention, Cambodia Office, Phnom Penh, Cambodia; 6 National Institute of Public Health, Phnom Penh, Cambodia; 7 WHO Collaborating Centre for Reference and Research on Influenza, Melbourne, Australia; 8 Armed Forces Research Institute of Medical Sciences, Bangkok, Thailand; Icahn School of Medicine at Mount Sinai, United States of America

## Abstract

**Background:**

The Cambodian National Influenza Center (NIC) monitored and characterized circulating influenza strains from 2009 to 2011.

**Methodology/Principal Findings:**

Sentinel and study sites collected nasopharyngeal specimens for diagnostic detection, virus isolation, antigenic characterization, sequencing and antiviral susceptibility analysis from patients who fulfilled case definitions for influenza-like illness, acute lower respiratory infections and event-based surveillance. Each year in Cambodia, influenza viruses were detected mainly from June to November, during the rainy season. Antigenic analysis show that A/H1N1pdm09 isolates belonged to the A/California/7/2009-like group. Circulating A/H3N2 strains were A/Brisbane/10/2007-like in 2009 before drifting to A/Perth/16/2009-like in 2010 and 2011. The Cambodian influenza B isolates from 2009 to 2011 all belonged to the B/Victoria lineage represented by the vaccine strains B/Brisbane/60/2008 and B/Malaysia/2506/2004. Sequences of the M2 gene obtained from representative 2009–2011 A/H3N2 and A/H1N1pdm09 strains all contained the S31N mutation associated with adamantanes resistance except for one A/H1N1pdm09 strain isolated in 2011 that lacked this mutation. No reduction in the susceptibility to neuraminidase inhibitors was observed among the influenza viruses circulating from 2009 to 2011. Phylogenetic analysis revealed that A/H3N2 strains clustered each year to a distinct group while most A/H1N1pdm09 isolates belonged to the S203T clade.

**Conclusions/Significance:**

In Cambodia, from 2009 to 2011, influenza activity occurred throughout the year with peak seasonality during the rainy season from June to November. Seasonal influenza epidemics were due to multiple genetically distinct viruses, even though all of the isolates were antigenically similar to the reference vaccine strains. The drug susceptibility profile of Cambodian influenza strains revealed that neuraminidase inhibitors would be the drug of choice for influenza treatment and chemoprophylaxis in Cambodia, as adamantanes are no longer expected to be effective.

## Background

Influenza is a major human pathogen associated with high morbidity and mortality, both in the temperate and subtropical/tropical regions. It is characterized by epidemics that occur seasonally throughout the world every year, with occasional pandemics arising from novel subtypes of the virus causing a considerable economic burden and significant cumulative morbidity and mortality [1]–[3]. Despite a plethora of information on influenza epidemiology and seasonality, which remains important in planning prevention and treatment strategies, overall patterns of infection have not been fully described on broad geographic scales and for specific types and subtypes of the influenza virus, thus highlighting the need for more countries to conduct year-round viral surveillance and report reliable incidence data at the type and subtype level, especially in the tropics [4].

In temperate regions influenza viruses typically circulate during the winter period [5]. In tropical areas, influenza activity usually occurs all year round with annual/biannual peaks in relation with rainy seasons and/or winter months, but infections can also occur without a clear seasonality [6]–[11]. Cambodia is a South-East Asian tropical country, which lies geographically in the Northern hemisphere but its influenza season occurs during June-December, each year [12]. Northern hemisphere countries usually experience influenza season from November to March/April, whereas the influenza season of southern hemisphere countries usually occur from May to September [13], [14]. Hence, Cambodia's influenza seasonality appears unusual and knowledge of the epidemiological and virological characteristics of such influenza circulation is important for public health preparedness.

We have previously reported preliminary data and described the circulation and seasonality of influenza viruses in Cambodia during three consecutive years following the establishment of the Cambodian National Influenza Centre (NIC) in 2006 [12]. In the present study, we documented the dynamics of influenza activity, performed antigenic and drug susceptibility analyses of influenza virus strains and conducted phylogenetic analysis of influenza A strains isolated between 2009 and 2011, which included the 2009 pandemic.

## Materials and Methods

### Geographic background

Cambodia is a tropical country of almost 15 million people, with a land area of 181,035 square kilometers in the southwestern part of the Indochina peninsula [15]. International borders are shared with Thailand and Laos on the West and the North, and Vietnam on the East and the Southeast. As the country is affected by monsoon, it is hot and humid with a mean temperature of 27°C and mean relative humidity of 77.5%. There are two distinct seasons: the dry season runs generally from November to April and the rainy season starts in May-June and ends in October-November.

### Patients

The Cambodian NIC was established in August 2006 as a joint collaboration between the Virology Unit at the Institut Pasteur in Cambodia (IPC), the Communicable Disease Control Department of the Ministry of Health (CDC/MoH) and the World Health Organization (WHO) office in Cambodia for the purpose of documenting the dynamics of influenza disease and to virologically characterize the circulating strains. To continuously monitor influenza activity, an outpatient sentinel surveillance system for influenza-like illness (ILI) with a weekly reporting and sampling scheme was initially established in five hospital sites in 2006. In addition, hospital-based surveillance of acute lower respiratory infection (ALRI) cases was established in two sites in Takeo and Kampong Cham provincial hospitals (SISEA project, French Agency for Development). Three additional ILI sentinel surveillance sites were then opened in 2009 and operated by the National Institute of Public Health (NIPH) in collaboration with the US CDC local office in the referral hospitals of Mondulkiri (Eastern Cambodia), Svayreang (South-East Cambodia) and Kampot (South-West Cambodia) Provinces. The Armed Forces Research Institute of Medical Sciences (AFRIMS) operated one sentinel site starting in 2009 in Western Cambodia in Thmor Kol (Battambang Province), and opened two additional sentinel sites in 2011 in Anlong Veng (Oddar Meancheay Province) and Pailin. During the pandemic of influenza A/H1N1 2009 virus, an event-based surveillance system was developed by CDC/MoH and WHO. The system was designed for rapid detection, reporting and confirmation of clusters of the disease or suspected cases admitted in both private clinics and hospitals [16].

An ILI case was defined by the sudden onset of fever (≥38°C axillary temperature) and cough or sore throat in the absence of other diagnosis. For the ALRI study, in children under 5, a suspect case was defined as an illness of <10 days duration with cough or breathing difficulties plus tachypnea. For the 5–14 years age group, case definition included the above symptoms plus fever (≥38°C axillary temperature) on admission. For patients over 15 years old, a case was defined as a person with fever (≥38°C axillary temperature) on admission plus tachypnea or chest pain or auscultatory crackles. For event-based surveillance of pandemic influenza A/H1N1 2009 virus (A/H1N1pdm09 virus) infection conducted from 2009 to 2010, inclusion criteria included any person with acute febrile respiratory illness (fever ≥38°C and respiratory symptoms, e.g. cough, sore throat, difficulty breathing) with no other apparent diagnosis and one or more of the following exposures to the risk of A/H1N1pdm09 virus infection within 7 days prior to symptoms onset: a) Close contact with a probable or confirmed case of A/H1N1pdm09 virus infection; b) Residing in or travelled to a province or foreign country with confirmed community transmission of A/H1N1pdm09 virus; c) Is part of a cluster of ILI cases; d) Handled specimens suspected of containing A/H1N1pdm09 virus.

An overview of the all of the surveillance and research programs involved with the collection and testing of strains for this study is provided in Table S1.

### Ethical statement

The ALRI study was approved by the National Ethics Committee of the Kingdom of Cambodia. All patients or parents of sick children who participated in the studies provided written informed consent. The ILI and event-based surveillance systems are public health activities organized by the Ministry of Health in Cambodia and as such have a standing authorization from the National Ethics Committee. Samples were all anonymized for the purpose of this study.

### Specimen collection

The sentinel sites obtained weekly epidemiological data from patients who fulfilled the ILI case definition, and collected naso-pharyngeal specimens from 5 to 10 cases per week. Respiratory samples together with clinical records were obtained from patients hospitalized with ALRI during 2009 and 2010 in Takeo and Kampong Cham hospitals. Naso-pharyngeal and throat specimens were placed in a vial containing 2 ml of virus transport medium (VTM). The specimens collected from the initial 5 sites of ILI sentinel surveillance and the 2 ALRI study sites were then immediately frozen in liquid nitrogen and the containers were sent on a weekly basis to the IPC's Virology Unit where specimens were stored at −80°C prior to testing. For the specimens collected from the 6 ILI sentinel surveillance sites newly established in 2009 and onward, the VTM tubes containing the naso-pharyngeal swab samples were stored at 4°C and shipped to the NIPH and/or AFRIMS laboratory for testing within 48 to 72 hours. For the A/H1N1pdm09 event-based surveillance, the specimens were collected mainly from hospitals and private clinics in and around the capital, placed into VTM, and immediately sent in a cool box to IPC's laboratory for testing.

### Laboratory methods

Viral RNA was extracted at the participating national laboratories (NIC/IPC, NIPH and AFRIMS) using commercial extraction kits (e.g. MagNa Pure LC, Qiagen Viral RNA mini kits) according to the manufacturer's instructions. RNA was amplified using real-time RT-PCR to detect influenza A and B viruses using standard protocols. Influenza A virus subtypes H1, H1pdm, H3 and H5 were detected using subtype-specific real-time RT-PCR assays. All samples that tested positive for influenza were sent to the NIC for confirmation and further characterization.

At the NIC's lab, viral RNA was extracted from 140 µL of virus transport medium containing the nasopharyngeal swab by using the MagNa Pure LC system (Roche), according to the manufacturer's instructions. Seasonal influenza viruses (A/H3N2, A/H1N1 and influenza B virus) were detected and subtyped by a multiplexed conventional reverse transcriptase-polymerase chain reaction (RT-PCR) as described previously [12]. The detection of A/H1N1pdm09 viruses was performed by using a one-step real-time RT-PCR according to the CDC protocol for detection and characterization of influenza A 2009 H1N1, with the use of a 96-well format IQ5 instrument (BioRad). Primer and probe reagents, reaction master-mix, and cycling parameters were used as described in the protocol [17]. The H5N1 virus was detected by a one-step real-time RT-PCR developed by the laboratory at the National Institute of Infectious Diseases (NIID), Tokyo, Japan, and recommended by WHO as the protocol for laboratory procedures to detect avian influenza A H5N1 virus in specimens from suspected human cases [18].

All of the influenza strains described in this study were isolated at the IPC laboratory by inoculation of the specimens that tested positive by molecular methods onto Madin-Darby canine kidney (MDCK) cells in a biosafety level 2+ laboratory for seasonal influenza viruses and in a biosafety level 3 laboratory for pandemic (H1N1) 2009 virus (until 2010, only) and for highly pathogenic avian influenza A/H5N1 virus. In brief, two hundred microlitres of each specimen was inoculated into MDCK cells, with 2 ml per well of maintenance medium containing TPCK trypsin (except for H5N1 virus) at a concentration of 2.0 µg/ml, in a 6-well plate. The plates were incubated at 35°C in a 5% CO_2_ atmosphere for 1 week to assess cytopathic effects. The influenza isolates were characterized by a hemagglutination inhibition assay using reference antigens and anti-sera kindly provided by the WHO Collaborating Center (WHOCC) for Reference and Research on Influenza in Melbourne, Australia. A representative number of influenza isolates collected by the various surveillance and event-based systems were sent each year to the WHOCC in Melbourne for confirmation and further analysis (Table 1).

**Table 1:** Influenza isolates collected in Cambodia by type and subtype in 2009-2011.

			Influenza Type		Subtype of Influenza A				WHOCC, Melbourne^a^		
Year	N# of specimens tested	N# of positives (%)	A (%)	B (%)	H1N1 (%)	H3N2 (%)	H5N1 (%)	H1N1pdm^b^ (%)	H1N1pdm^b^	H3N2	IB^c^
2009	4601	747 (16.2)	653 (87.4)	94 (12.6)	2 (0.3)	266 (40.7)	1 (0.2)	384 (58.8)	10	15	15
2010	3507	446 (12.7)	343 (76.9)	103 (23.1)	0 (0)	155 (45.2)	1 (0.3)	187 (54.5)	31	17	41
2011	2705	491 (18.2)	173 (35.2)	318 (64.8)	0 (0)	44 (25.4)	8 (4.6)	121 (70)	36	14	72

a Specimens analyzed by the WHO Collaborating Center in Melbourne from 2009 to 2011.

b H1N1pdm09.

c Influenza B.

### Genome sequencing and phylogenetic analysis

At the NIC's laboratory, viral RNA was extracted from 200 µl of MDCK supernatant using the QIAamp Viral RNA Mini Kit, according to the manufacturer's recommendations (Qiagen, Hilden, Germany). RNA was used as template for RT-PCR with subtype and segment-specific primers (reaction conditions and primer sequences are available from the authors upon request). Nucleotide sequencing reactions were performed with BigDye Terminator Cycle Sequencing kits (Applied Biosystems) following the protocols supplied by the manufacturer and the products were sequenced using an ABI 3730XL automatic DNA Analyser (Life Technologies, Carlsbad, CA, USA) in a commercial facility (Macrogen, Seoul, Korea). Multiple sequence alignment was conducted using ClustalW, version 2 [19]. Phylogenetic trees based on the HA gene sequences were obtained by using the Neighbor-joining method, HKY model, generated using PAUP software [20]. Bootstrap analysis (*n* = 1000) was carried out to determined the best-fitting tree. Vaccine strain sequences, as well as sequences from viruses collected in other countries were obtained from EpiFlu Database available via the GISAID website (www.gisaid.org), and were included in the analysis.

### Nucleotide sequence accession numbers

All Cambodian influenza A/H3N2, A/H1N1pdm09 and Influenza B virus sequences included in the analysis were submitted to Genbank and/or to EpiFlu Database and all of these sequences are available via GISAID website (www.gisaid.org). Table S2 provides detailed information about all of the Cambodian strains and sequences analysed in this study.

### Neuraminidase inhibitors susceptibility assays

Representative isolates, with respect to date of detection and province of origin of the patient, were randomly selected for neuraminidase drug testing. The NA-Star kit (Life Technologies, Carlsbad, CA, USA), a chemiluminescent NA inhibition assay which utilizes a 1,2-dioxetane derivative of sialic acid as substrate, was used according to the manufacturer's instructions. Oseltamivir carboxylate, zanamivir and 4-amino-4-deoxy-Neu5Ac2en were provided by the Institute for Glycomics, Griffith University, Gold Coast, Australia. Compounds were prepared in distilled water and stored at −20°C until time of use. The concentration of drug required to inhibit 50% of the NA activity (IC50) was calculated using the non-linear curve-fitting function in the Graphpad Prism 4 package (GraphPad Software, Inc., La Jolla, CA, USA). The average IC50 (nM) (± standard deviation) of two independent determinations was calculated for each virus. Outliers of more than 2 standard deviations from the overall mean were retested twice [21].

### Susceptibility to adamantanes

The full M gene segments from representative influenza strains of the influenza A/H3N2, A/H1N1 and A/H1N1pdm09 subtypes virus strains isolated from humans were sequenced to detect mutations associated with resistance to adamantanes. Amino acids at positions 26, 27, 30 and 31 of the transmembrane region of the M2 protein are the residues most frequently associated with amantadine- and rimantadine-resistant strains.

### Statistical analysis

The comparisons between percentages and two means were tested by chi-squared test and Student's t test respectively. A p value <0.05 was considered statistically significant. Proportions, means and all statistical analyses were performed using STATA 9.0 (StataCorp., College Station, TX, Texas).

## Results

### Influenza activity in Cambodia

The Cambodian ILI surveillance program collected a total of 7376 samples during 2009–2011. The average age of the ILI patients was 8.7 years (range, 2 weeks to 84 years) and 51.8% were male. Of these, 1246 (16.9%) tested positive for influenza virus: 17.6% in 2009, 14.5% in 2010 and 18.7% in 2011. The average age of influenza virus-infected patients was 7.3 years (range, 1 month to 65 years) and 53.9% were male. There was a statistical difference between the age of confirmed influenza-positive patients and those who tested negative (7.3 versus 8.9 years, p<0.001).

During 2009–2010, a total of 2248 patients presenting with ALRI symptoms were tested. Their mean age was 34.6 years (range, 3 weeks to 95 years) and 52% were male. Of these, 59 (2.6%) tested positive for influenza virus (3% in 2009 and 1.5% in 2010). The median age of influenza-positive cases among ALRI was 16.9 years (range, 3 months to 70 years), and 40.7% were male. The patients with influenza virus infection were significantly younger than the patients who tested negative in the ALRI study (16.9 versus 35 years, p<0.001).

During 2009 and 2010, of the 1005 patients identified through event-based surveillance for suspected A/H1N1pdm09 virus infection 347 (34.5%) were positive for A/H1N1 2009 virus by real-time RT-PCR.

**Figure 1:**
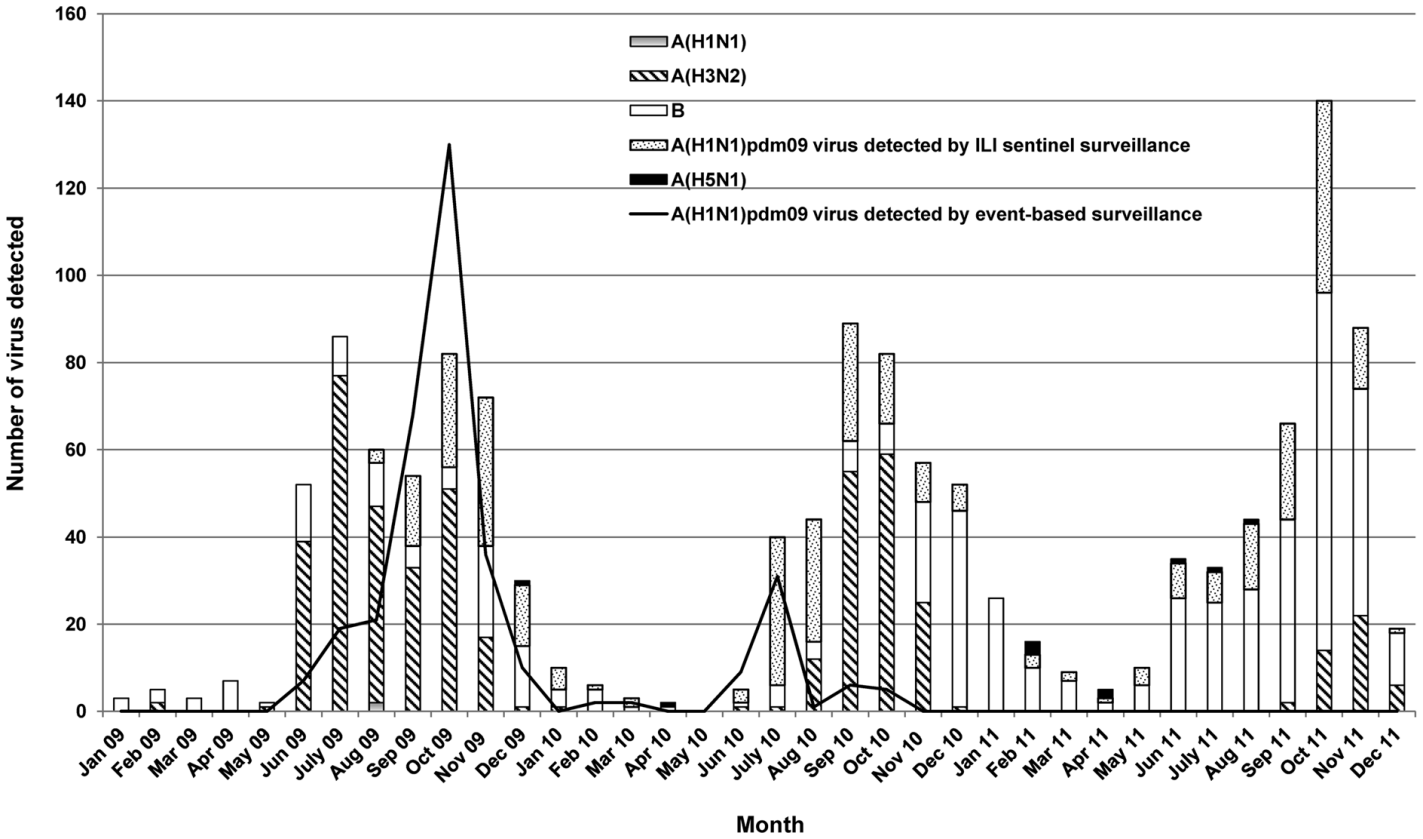
Monthly distribution of influenza virus detected from 2009 to 2011.

From 2009 to 2011, influenza activity was observed mainly from June to December. The proportion of influenza virus-positive samples detected per month among ILI specimens between 2009 and 2011 varied from 0 to 21% during the dry seasons and from 3 to 61% during the rainy months, from June to November (Figure S1). Only 6 to 12% of the ALRI specimens were positive for influenza virus during the transmission season (Figure S1). The total number of human influenza strains detected during the 2009–2011 period by ILI, ALRI and A/H1N1pdm09 virus event-based surveillance are detailed in Table 1 and Figure 1.

**Figure 2:**
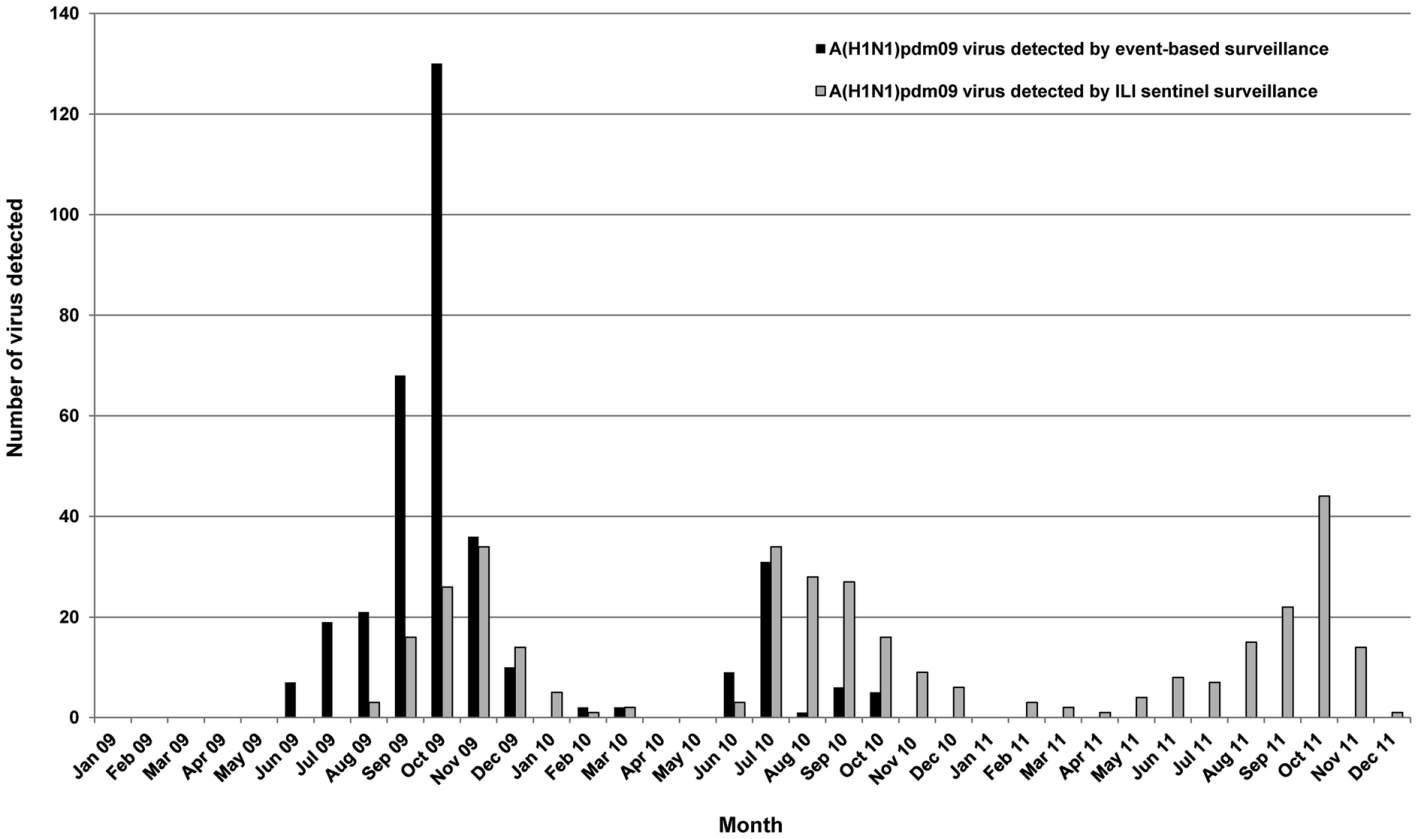
Monthly distribution of H1N1pdm09 virus detected from 2009 to 2011.

The A/H1N1pdm09 virus was first detected on June 23^rd^ 2009 and progressively became, along with H3N2, the most frequent influenza A subtype circulating in Cambodia. A/H1N1pdm09 activity, detected via event-based surveillance, demonstrated a peak during the period from September to November 2009, dropping to a very low level between January and May 2010 before increasing again to a small peak in June-July 2010. Community transmission of this virus began in August 2009, after a 2 month-period during which only imported cases were identified. The peak of incidence corresponded to the spread of the virus across the country when it started to be detected by both the ILI and the event-based surveillance systems (Figure 2). A/H3N2 virus was frequently isolated in 2009 and 2010 but in 2011 the incidence of this virus was lower.

Influenza B virus co-circulated with A/H3N2 and A/H1N1pdm09 viruses during the 2009–2011 period, with the peak detection in the rainy season of 2011.

### Antigenic analysis

Antigenic analyses indicated that A/H3N2 strains belonged to the A/Brisbane/10/2007-like group in 2009 before drifting to the A/Perth/16/2009-like group in 2010 and 2011. All A/H1N1pdm09 strains detected from 2009 to 2011 belonged to the A/California/7/2009-like group. The influenza B strains isolated in 2009–2011 demonstrated the antigenic characteristics of the B/Brisbane/60/2008-like and B/Malaysia/2506/2004 viruses (B/Victoria/2/87 lineage) (Table 2).

**Table 2:** Comparison between vaccine strains and circulating influenza strains in Cambodia from 2009 to 2011.

	A/H1N1pdm09		A/H3N2		Influenza B	
Season	Vaccine strains	Cambodian strains	Vaccine strains	Cambodian strains	Vaccine strains	Cambodian strains
**2009**	**A/California/7/2009-like**	**A/California/7/2009-like**	**A/Brisbane/10/2007-like**	**A/Brisbane/10/2007-like**	**B/Florida/4/2006-like**	**B/Brisbane/60/2008-like**
*2009*–*2010*	*A/California/7/2009-like*		*A/Brisbane/10/2007-like*		*B/Brisbane/60/2008-like*	
**2010**	**A/California/7/2009-like**	**A/California/7/2009-like**	**A/Perth/16/2009-like**	**A/Perth/16/2009-like**	**B/Brisbane/60/2008-like**	**B/Brisbane/60/2008-like; B/Malaysia/2506/2004-like**
*2010*–*2011*	*A/California/7/2009-like*		*A/Perth/16/2009-like*		*B/Brisbane/60/2008-like*	
**2011**	**A/California/7/2009-like**	**A/California/7/2009-like**	**A/Perth/16/2009-like**	**A/Perth/16/2009-like**	**B/Brisbane/60/2008-like**	**B/Brisbane/60/2008-like; B/Malaysia/2506/2004-like**
*2011*–*2012*	*A/California/7/2009-like*		*A/Perth/16/2009-like*		*B/Brisbane/60/2008-like*	

### Antiviral drug resistance

Among the influenza strains collected during 2009–2011, all 34 A/H3N2 and 39 of 40 (97.5%) A/H1N1pdm09 isolates showed genetic markers of resistance to adamantanes (Table S3). All of the isolated amantadine-resistant viruses contained the amino acid change from serine to asparagine at position 31 (Ser31Asn) in the M2 protein. All influenza A viruses that were sequenced indicated resistance to adamantanes, except for one A/H1N1pdm09 strain isolated in 2011. Analysis of susceptibility to the neuraminidase inhibitors demonstrated that all of the tested strains were sensitive to oseltamivir and zanamivir (Table 3).

**Table 3:** Neuraminidase inhibitors resistance in influenza viruses, 2009–2011.

	Resistance to neuraminidase inhibitors			
	Oseltamivir		Zanamivir	
Virus type and subtype	Isolates tested	Resistant N# (%)	Isolates tested	Resistant N# (%)
A/H3N2	12	0	12	0
A/H1N1pdm09	61	0	61	0
B	81	0	81	0

### Molecular characterization of A/H3N2 isolates

**Figure 3:**
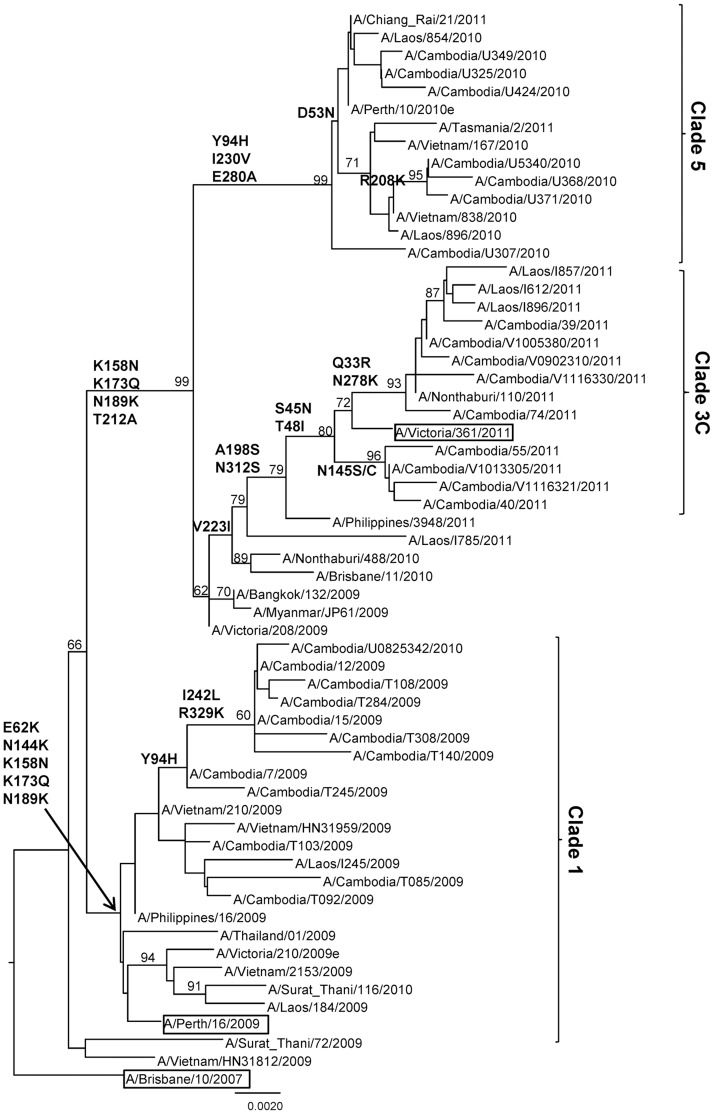
Phylogenetic analysis of the HA1 domains of the HA genes (sequences of 987 nucleotides (nt49–1035)) of influenza A/H3N2 virus isolates collected in Cambodia from 2009 to 2011. The phylogenetic analysis was conducted as a distance-based neighbour-joining phylogenetic tree of influenza using the HKY model and generated using PAUP software with 1,000 bootstrap replicates (values ≥60 shown on branch) and rooted to A/Brisbane/10/2007. Major amino acid changes are shown in block letters at the appropriate nodes. The vaccine reference strains are boxed. Scale bar indicates number of nucleotide substitution per site.

Phylogenetic analysis was carried out for the sequences of the HA1 domain of 28 representative A/H3N2 strains isolated from 2009 to 2011 in Cambodia. Additional sequences retrieved from GenBank and/or from EpiFlu Database (via GISAID) and corresponding to vaccine seed strains, to viruses isolated in Australia and New Zealand (southern hemisphere), and various other South-East Asian countries (Vietnam, Laos, Thailand, Myanmar, and Philippines) were included in the analysis. As shown in Figure 3 (Table S4 and Figure S2), the HA sequences of the A/H3N2 viruses isolated during the three consecutive seasons fell into three distinct clusters also corresponding to strains detected during distinct influenza seasons: Cluster 1 (clade 1) with virus isolated in 2009 and with one strain detected in August 2010 (A/Cambodia/U0825342/2010); cluster 2 (clade 5) with isolates obtained in 2010; cluster 3 (clade 3C) with isolates obtained in 2011. The viruses from the cluster 1 belonged to the A/Perth/16/2009 clade 1 viruses and contained five characteristic amino acid changes, E62K, N144K, K158N, K173Q and N189K compared to that of A/Brisbane/10/2007 virus. The viruses from clusters 2 and 3 had multiple amino acid changes in common compared to that of the A/Brisbane/10/2007 virus, namely K158N, K173Q, N189K and T212A. The Cambodian A/H3N2 viruses isolated in 2010 and 2011 from these clusters diverged into two major genetic clades represented by A/Perth/10/2010 and A/Victoria/361/2011, respectively. The A/Perth/10/2010 clade 5 viruses had characteristic mutations, Y94H, I230V and E280A. The 2011 A/H3N2 Cambodian isolates belonged to clade 3C represented by A/Victoria/361/2011 defined by the mutations S45N, T48I, A198S, V223I and N312S.

Isolates from each group were interspersed with strains isolated from other tropical countries throughout the phylogenetic groups in which the strains from Australia (southern hemisphere) were clearly positioned basal to each viral clade.

### Molecular characterization of A/H1N1pdm09 isolates

**Figure 4:**
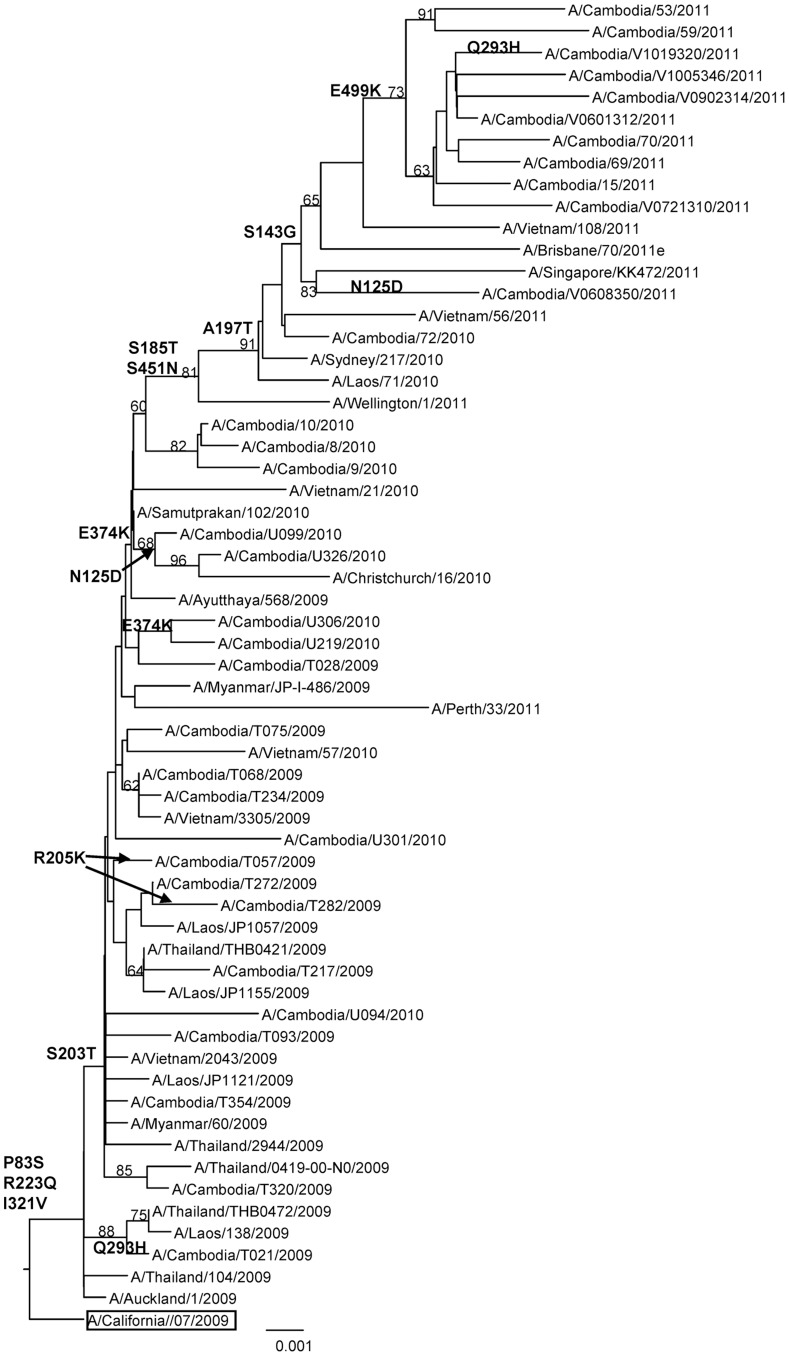
Phylogenetic analysis of the HA genes (1685nt (nt1–1685)) of influenza A/H1N1pdm09 virus isolates collected in Cambodia from 2009 to 2011. The phylogenetic analysis was conducted as a distance-based neighbour-joining phylogenetic tree of influenza using the HKY model and generated using PAUP software with 1,000 bootstrap replicates (values ≥60 shown on branch) and rooted to A/California/07/2009. Major amino acid changes are shown in block letter at the appropriate nodes. The vaccine strain is boxed. Scale bar indicates number of nucleotide substitution per site.

In order to gain insight into the degree of genetic variability of A/H1N1pdm09 isolates in Cambodia, a phylogenetic tree analysis was carried out for 33 HA sequences from A/H1N1pdm09 strains circulating from 2009 to 2011, comprising the region encoding the HA1 and HA2 domain of the HA protein. The trees were created with the corresponding regions of 21 HA sequences from strains isolated from various other South-East Asian countries (Vietnam, Laos, Thailand, Myanmar, and Singapore). As seen in Figure 4 almost all of the Cambodian isolates fell into the S203T clade. Although one of the Cambodian strains isolated in mid-July 2009 (A/Cambodia/T021/2009) did not cluster with this clade. The majority of strains collected in 2010 and all strains collected in 2011 shared an amino acid change at position E374K. Within this group, one subgroup of strains detected in 2010 shared an amino acid change at position N125D and another subgroup of strains collected in 2011 had the characteristic mutations S143G, S185T, A197T, S451N and E499K. The amino acid substitutions are outlined in detail in Figure 4 (Table S5 and Figure S3).

Compared to the vaccine strain A/California/07/2009 all of the Cambodian isolates possessed residues D187 and D222 in the receptor-binding site. The most common variations compared to the vaccine strain were P83S, S203T, R223Q and I321V, which were actually the substitutions defining clades. These mutations were observed in all Cambodian isolates except for A/Cambodia/T021/2009, which did not possess the amino acid change at position S203T. However, this strain possessed the substitution Q293H. This mutation was also detected in one strain in 2011 (A/Cambodia/V1019320/2011). Variations were also observed on viral HA antigenic sites. Two of the Cambodian isolates, A/Cambodia/T282/2009 and A/Cambodia/T057/2009 possessed R205K substitutions at the Ca1 antigenic site of the haemagglutinin. Moreover, some of the Cambodian strains isolated in 2010 and one strain in 2011 contained the substitutions N125D involving the Sa haemagglutinin antigenic site. Substitutions in the HA2 domain of the HA protein were also observed, particularly the E374K, in the majority of Cambodian strains isolated in 2010 and in strains isolated in 2011.

### Molecular characterization of influenza B isolates

**Figure 5:**
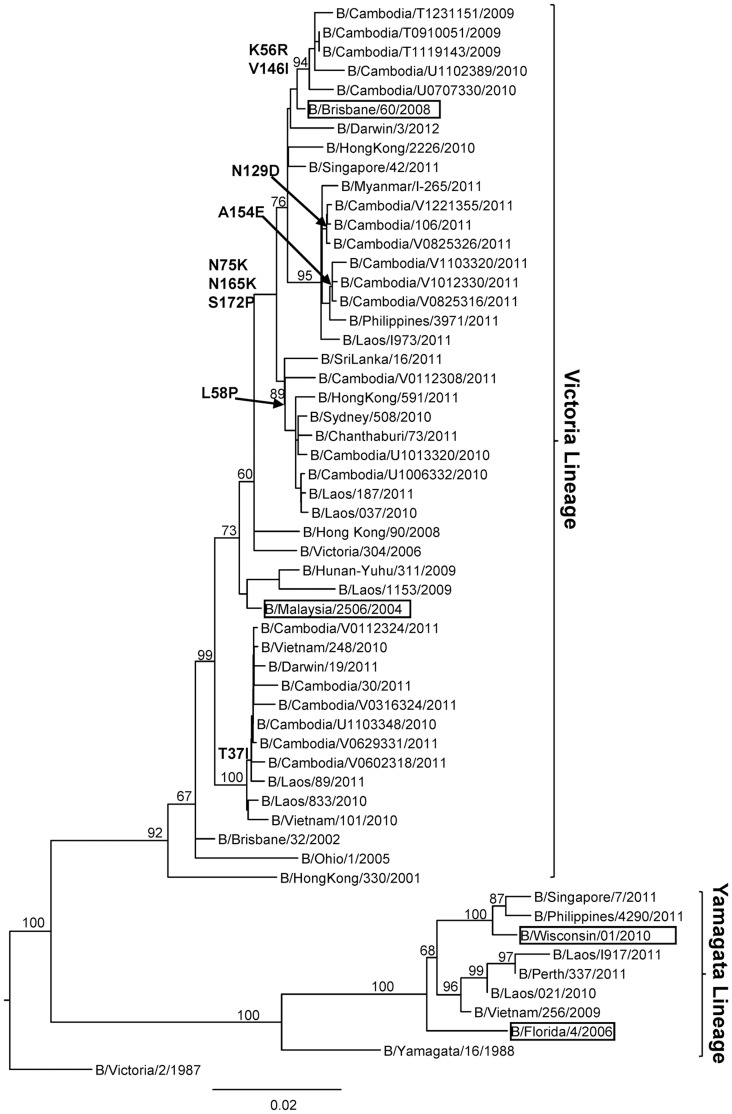
Phylogenetic analysis of the HA1 domains of the HA genes (sequences of 1041 nucleotides (nt 46–1086)) of influenza B virus isolates collected in Cambodia from 2009 to 2011. The phylogenetic analysis was conducted as a distance-based neighbour-joining phylogenetic tree of influenza using the HKY model and generated using PAUP software with 1,000 bootstrap replicates (values ≥60 shown on branch) and rooted to B/Victoria/2/1987. Major amino acid changes are shown in block letters at the appropriate nodes. The vaccine reference strains are boxed. Scale bar indicates number of nucleotide substitution per site.

The results of the phylogenetic analyses of the HA1 sequence data generated from 20 representative Cambodian influenza B viruses obtained from 2009 to 2011 are shown in Figure 5. It can be clearly seen that Cambodian influenza B isolates in 2009 to 2011 belonged to the B/Victoria-lineage represented by the vaccine strain B/Brisbane/60/2008 and B/Malaysia/2506/2004. All of the 2009 isolates, most of the 2010 isolates and more than two thirds of the 2011 isolates belonged to genetic group 1 (including influenza B vaccine strain B/Brisbane/60/2008), sharing amino acid changes at positions N75K, N165K and S172P. There were three subgroups within genetic group 1: one subgroup with isolates collected in 2009 and 2010, shared amino acid changes at K56R and V146I in the HA; another subgroup of strains isolated in 2011 (which had two different subgroups with mutation N129D and A154E respectively); and the other subgroup with some of the isolates collected in 2010 and one strain in 2011, shared an amino acid change at L58P in the HA. Some other viruses collected in 2011 and one 2010 isolate belonged to genetic group 2 sharing an amino acid change T37I which was a specific mutation of genetic group 2. No influenza B viruses isolated between 2009 and 2011 belonged to the B/Yamagata-lineage represented by vaccine strain B/Wisconsin/01/2010.

Regions of the Cambodian influenza B virus isolates containing 347 amino acids of HA were compared with those of reference vaccine strains in order to understand the variations within the Victoria lineage of influenza B viruses (Table S4). The Victoria lineage strains were divided into two distinct groups, B/Malaysia/2506/2004 and B/Brisbane/60/2008 (Table S4). All Cambodian influenza B viruses isolated between 2009 and 2011 presented in this study and the vaccine strain B/Brisbane/60/2008 had two amino acid changes (S134P and A199T) compared to B/Malaysia/2506/2004. The B viruses isolated from 2009, 2010 and 2011, which belong to genetic group 1 of Victoria lineage, had three common amino acid changes from B/Malaysia/2506/2004 at N75K, N165K and S172P in their HA genes. In this genetic group 1 of Cambodian influenza B viruses, the subgroup of isolates in 2009 and 2010, had amino acid changes at K56R and V146I. The subgroup of strains isolated in 2011 had two different subgroups with the mutations N129D and A154E respectively. The other subgroup, containing some of the isolates collected in 2010 and 1 strain of 2011, shared an amino acid change at L58P in the HA. The other amino acid changes of this genetic group 1, compared to the B/Malaysia/2506/2004 strain, occurred at I7V for the B/Cambodia/T1231151/2009 strain, K162N for the B/Cambodia/U0707330/2010 strain, S255P for the B/Cambodia/V1103320/2011 strain and K345R for the B/Cambodia/U1102389/2010 strain. Some other isolates from 2011 and one isolate from 2010 were clustered together in genetic group 2 of Victoria lineage. These strains had one common amino acid change from B/Malaysia/2506/2004 at T37I; and variation occurring at P31S and N197S for the B/Cambodia/30/2011 strain; and at V90I for the B/Cambodia/V0316324/2011 strain. No Cambodian B viruses isolated from 2009–2011 belonged to the Yamagata lineage (Figure 5, Table S6 and Figure S4).

## Discussion

Seasonal influenza virus epidemics occur in temperate regions of the world each winter, from November to March in the northern hemisphere and from May to September in the southern hemisphere [27]. Although influenza virus has long been regarded as a “cold-weather” pathogen due to its marked winter epidemics in the temperate zones, recent studies show that tropical regions can have significant year-round influenza virus activity [5], [8], [11], [13], [28], [29]. In contrast, this study combined with our previous analysis of influenza activity in Cambodia from 2006 to 2008 has demonstrated that the seasonality of influenza in Cambodia, which is located in the northern hemisphere, has a consistent pattern characteristic of influenza circulation in the southern hemisphere, peaking during the rainy season from June to November as we previously reported [12]. Cambodian influenza activity reveals a discrete peak for influenza A during June-December, whereas year round circulation of influenza B and rare cases of H5N1 and other influenza A viruses occur during the dry season. This pattern is intermediate between southern hemisphere and tropical transmission.

We found no statistically significant sex ratio difference between non-influenza and influenza infected patients. As previously reported in our study from 2006 to 2008 [12], the proportion of severe influenza infections detected among hospitalized patients with ALRI from 2009 to 2010 during the influenza season was lower compared to the number of patients positive for influenza virus observed in the ILI surveillance system (Figure S1). During 2009–2010 periods, only 2.6% of the patients recruited in ALRI surveillance were identified as positive for influenza virus infection, whereas 16.9% were observed among patients within the NIC's ILI surveillance. This low frequency of severe influenza cases, which was also found in another Cambodian study [22] could be related to yearly variations in influenza severity [23]; however, the true burden of severe influenza is likely to be underestimated. Although the cases detected were from hospital-based surveillance, there might be an unknown proportion of cases that were missed due to poor access to hospital care. This is particularly an issue in developing countries such as Cambodia [23]. Furthermore, some studies have shown that influenza viruses predispose to secondary bacterial infections [24], [25] and we cannot exclude that bacterial lung infections diagnosed among patients recruited in our ALRI study were not associated with initial influenza disease. In developing countries such as Cambodia poor access to healthcare may result in late presentation to hospitals and clinics when influenza is no longer detectable in clinical specimens. To rule out this hypothesis, serological testing for influenza exposure is required among patients with bacterial respiratory infections during the seasonal influenza epidemics. The A/H1N1pdm09 virus infection detected both in event-based surveillance and sentinel-based surveillance systems occurred in all age groups. However, the median ages of event-based patients tended to be older than that of influenza positive patients detected in ILI surveillance. This finding is consistent with the characteristics of hospitalized cases observed in the USA [26].

The first imported A/H1N1pdm09 cases from Cambodia were detected from June to July 2009 through event-based surveillance in two areas where major international border crossings take place: the international air travel through the Phnom Penh International Airport and the international overland border crossings from Poipet, Thailand to Siem Reap Province. Positive cases of this virus were thereafter detected from communities around the capital and from the ILI sentinel sites in Siem Reap and Battambang Province by both event-based and sentinel surveillance from August 2009. In October 2009, the virus spread throughout the country as indicated by ILI and ALRI surveillance data. The number of A/H1N1pdm09 virus isolates detected by the event-based surveillance compared to the number detected by the sentinel (ILI and ALRI) surveillance systems during the highest point of the outbreak in October 2009 was 5∶1 (Figure 2), suggesting that when it comes to the timely detection of outbreaks and important public health events, event-based surveillance is more sensitive than sentinel surveillance systems, which are not suited to the detection of rare but high-impact outbreaks of emerging and unknown diseases [16]. However, at the start of the influenza season in June 2010, the A/H1N1pdm09 isolates were first detected by both the event-based and sentinel surveillance systems before being mostly detected by the sentinel surveillance system for the rest of the season. This finding is consistent with the WHO's declaration on 10 August 2010 that the World was moving into the post-pandemic period and confirmed that this virus was taking on the behavior of a seasonal influenza virus [30].

Worldwide, there continued to be co-circulation of influenza A/H1N1pdm09, A/H3N2 and B viruses in 2010, with the latter two being predominant [31]. In Cambodia, since September 2009, there was a sustained circulation of predominantly A/H1N1pdm09 viruses, but also to a lesser extent, seasonal influenza H3N2 and B virus, with the latter being the most frequently detected strain from November and December 2010 through 2011. During this period, limited data from Vietnam indicated community transmission of influenza there as well, predominantly influenza type B, but the remainder of southern Asia including India, Bangladesh, Thailand, Singapore, China Hong Kong Special Administrative Region, Southern China and Chinese Taipei reported small numbers of a mixture of all three circulating types [32]. The circulation of type B virus tends to rise as overall influenza activity declines. There was a surge of influenza B cases near the end of each influenza season, usually from November to December, as shown in our previous report from 2006 to 2008 [12]. There are frequent bottleneck years during which prevalence of B strains is low, which usually was correlated with high prevalence of influenza A strains. Indeed, low level of B viruses in the year 2009 was observed during the pandemic of A/H1N1pdm09 virus. Once the bottleneck is relieved, there are usually changes in the prevalence of B strains which could explain the rise of influenza B virus at the end of 2010 and in 2011 after the A/H1N1pdm09 subsided (Figure 1). From 2007 to 2011, except in 2009, the influenza B strains identified in Cambodia were well matched by the recommended influenza B vaccine virus (Table 2) [12]. Currently, seasonal influenza vaccines are only available through private market purchase and Cambodians are not accustomed to influenza vaccination [33]. However, it could be problematic if the B strain that was circulating did not match the one contained in the seasonal influenza vaccine. Mismatches between influenza B strains circulating in Cambodia and strains included in the trivalent vaccine should lead to the use of a quadrivalent vaccine that contains both influenza B lineages.

The emergence of resistance following treatment with amantadine has occurred only transiently and the levels of circulating resistant viruses were low until 2004 [34], [35]. In Cambodia, no amantadine-resistant strains were found in seasonal influenza viruses in 2006, but the prevalence increased to 100% in 2007 and was maintained at this level through 2011 [12]. This finding supports that of a study conducted by another group reporting that adamantane resistance in influenza A viruses increased in 2007 in South East Asia [36]. Surveillance for antiviral susceptibility of influenza viruses in the Asia-Pacific region during 2011, showed that >99% of influenza A strains continued to be resistant to the adamantane drugs [37]. The high levels of amantadine resistance in influenza A virus strains in the community were not related to domestic amantadine use, as antiviral drugs for upper respiratory infection is uncommon among Cambodians. Following the outbreaks of severe acute respiratory syndrome and avian influenza, an unusually high prevalence of amantadine resistance has been observed most prominently in China, Hong Kong, and Taiwan since 2003, probably due to widespread use of amantadine [34], [38]. However, there was also evidence that the spread of resistant A/H3N2 virus was related to linked fitness mutations and not related to drug pressure [39], [40]. Cambodia's rise of resistance in 2007 may therefore reflect a strong influence from China or Taiwan in terms of influenza transmission.

A single Cambodian A/H1N1pdm09 strain, isolated from a 10 year old girl patient in Takeo Province in mid-May 2011, did not contain the mutation Ser31Asn nor any of the transmembrane region of the M2 protein mutations known to be associated with adamantane resistance [34].

Alongside neuraminidase inhibitors, oseltamivir and zanamivir have never been used to any large extent in the country and there is no evidence that any of the Cambodian patients were exposed to the drugs before or during influenza infection. The general lack of primary resistance to this class of antiviral has been previously reported before introduction of the neuraminidase inhibitors anywhere in the world [41]. In 2008, a low prevalence of neuraminidase inhibitors resistance (<1%) was detected in isolates from untreated patients [42]. During 2011, neuraminidase inhibitors resistance in A/H1N1pdm09, A/H3N2 and B viruses in the Asia-Pacific region remained low (<3% and <1% for influenza A and B strains respectively) [37]. Therefore, it is not surprising that all Cambodian isolates tested retained sensitivity to oseltamivir and zanamivir. However, in our previous study, we detected two influenza A/H1N1 strains with the H275Y mutation, associated with a high level of resistance to oseltamivir [43]. One of these strains detected in October 2007 showed high resistance to neuraminidase inhibitors by antiviral drug susceptibility assay, even though the other strain could not be grown in Madin-Darby canine kidney cells. Following the emergence in Europe in late 2007 of a transmissible human A/H1N1 influenza strain that was highly resistant to the neuraminidase inhibitor oseltamivir, the emergence of similar viruses was observed in South Africa and several countries in Oceania and South East Asia, especially from May 2008 onwards [44]. However, the mutant H275Y virus found in Cambodia is not a variant of the emergent H275Y strain detected elsewhere, but instead has arisen independently, as occasionally reported previously [42], [45].

The phylogenetic analysis of the HA1 genes of A/H3N2 strains from Cambodia showed three groups corresponding to three consecutive influenza seasons from 2009 to 2011. Each group, except the group from 2010–2011, belonged to the phylogenetic group represented by the corresponding vaccine strain. We previously demonstrated that there was co-circulation of different lineages of viruses isolated in 2005 and probable re-emergence of A/H3N2 viruses in the two consecutive seasons of 2005–2006 [42]. The HA sequences from the A/H3N2 isolates from 2009 to 2011 fell within three distinct phylogenetic clades. These findings are consistent with the dynamic evolution of A/H3N2 viruses that present a characteristic co-circulation of several variants for up to three years, which are subsequently replaced by new emerging variants with different antigenic features. This constant evolution of A/H3N2 viruses leads to the emergence of new viral variants that can elude the human immune response, causing outbreaks [46], [47]. This viral reintroduction plays a key role in the genesis of new clades and the global spread of these novel influenza virus variants. The Cambodian isolates collected from each season seem to have evolved from common ancestor isolates that are also probably at the origin of Australian srains [43], [48]. Additional research is required to more precisely define factors that contribute to this influenza seasonality in Cambodia such as the role of climate in triggering seasonal epidemics and the role for natural selection and host susceptibility.

The earliest A/H1N1pdm09 isolates from Cambodia (A/Cambodia/T028/2009, A/Cambodia/T075/2009, A/Cambodia/T272/2009, A/Cambodia/T282/2009, A/Cambodia/T093/2009 and A/Cambodia/T021/2009) were detected in the period from the end of June to mid-July 2009 through event-based surveillance focused on American students visiting Phnom Penh, the capital city of Cambodia. Positive cases of this virus were thereafter detected from communities all over the country from August 2009 by both the event-based and sentinel surveillance systems. Except for A/Cambodia/T021/2009, all of the other strains detected in that period clustered into the S203T clade. This finding suggested that the A/H1N1pdm09 viruses circulating in Cambodia in that period were introduced into the country, possibly from the USA. Since August 2009, all strains isolated in 2009, 2010 and 2011 were assigned to this clade. This is in agreement with recent results obtained in other regions of the world where it was the most commonly detected clade [49], [50].

All of the Cambodian isolates possessed residues D187 and D222 in the receptor-binding site, which is known to confer binding of H1 viruses to the human receptor, supporting efficient transmission of these viruses in humans [51]. In other studies the D222G substitution was detected more frequently in viruses isolated from patients with fatal outcomes and in isolates from lungs [50], [52]. This residue was well conserved in all Cambodian strains isolated from patients, but no fatal cases were examined in these studies. The substitution Q293H had been observed recently in a substantial proportion of virus strains isolated from postmortem samples from patients who died from laboratory-confirmed cases of A/H1N1pdm09 infection [52]. Two Cambodian isolates (A/Cambodia/T021/2009 and A/Cambodia/V1019320/2011) possessed this mutation and caused pneumonia requiring hospital admission, although there was no fatal complication. The known antigenic sites in the HA of the 1918 pandemic virus have been conserved in the pandemic A/H1N1pdm09 virus [53]. Molecular analysis showed that these sites were relatively conserved in Cambodian isolates. Two isolates, A/Cambodia/T282/2009 and A/Cambodia/T057/2009, which caused mild symptoms, possessed the R205K variation at the Ca1 antigenic site of the hemagglutinin without altering the properties of the polar and basic amino acid. The other two isolates from 2010 (A/Cambodia/U099/2010 and A/Cambodia/U326/2010) and one isolate from 2011 (A/Cambodia/V0608350/2011), which caused severe illness, had a substitution N125D at the hemagglutinin Sa antigenic site, which slightly modified the properties of the neutral side chain to a basic one. Immunological pressure may have driven virus evolution as shown by the displacement of E374E by E374K, a site important for membrane fusion, and the emergence of the two R205K and N125D amino acid substitutions involving the antigenic sites Ca1 and Sa respectively [54]. These antigenic sites contain many amino acids involved in neutralising epitopes near the receptor-binding pockets [53]. Although a single amino acid substitution involving one antigenic site may be sufficient to cause antigenic change, more commonly antigenic drift variants of epidemiological importance have resulted from changes of at least four amino acids across two or more antigenic sites [55]. Thus the variations observed in the hemagglutinin region of Cambodian A/H1N1pdm09 virus isolates most likely did not affect the antigenicity of the viruses. Although the results from antigenic analysis of Cambodian strains support this hypothesis, *in vitro* results cannot always predict *in vivo* effects.

There have been two distinct evolutionary lineages of influenza B viruses in recent years, represented by the two epidemic strains, B/Victoria/2/87 and B/Yamagata/16/1988. Phylogenetic analyses of influenza B isolates from Cambodia indicated that the circulating influenza B viruses in 2009 to 2011 belonged to the Victoria lineage; and the Yamagata lineage viruses did not circulate in Cambodia during those years (Figure 5). Some strains from 2010 and 2011 were closely related to the vaccine reference strain B/Malaysia/2506/2004, whereas the remainder of strains from 2009–2011 were closely related to the vaccine strain B/Brisbane/60/2008 (Figure 5 and Table S4). Our results indicated that a mismatch between the vaccine and epidemic strains in Cambodia occurred in the Southern Hemisphere World Health Organization 2009 influenza B virus vaccine recommendations; B/Brisbane/60/2008-like should have been the candidate strain for Cambodia (Table 2). Interestingly, the influenza B viruses that circulated in 2010 were found to have evolved from different genetic groups, indicating that the outbreak was caused by multiple sources. All of the influenza B viruses collected in 2009 and the majority of the 2010 and 2011 viruses possessed amino acid substitutions N75K, N165K and S172P that defined genetic group 1 in which the vaccine B/Brisbane/60/2008 strain was grouped. All of the 2009 and some of the 2010 viruses carried the amino acid substitution K56R compared with the vaccine B/Brisbane/60/2008 strain; some strains from 2010–2011 viruses carried the substitution T37I and L58P; some isolates from 2010 and all strains from 2011 contained amino acid I146V compared with the vaccine B/Brisbane/60/2008. None of these substitutions had a marked effect on the antigenicity of the viruses. This is not unusual, as influenza B virus activity is highly variable world-wide, with no established pattern [56]. However, this could lead to the emergence of influenza B reassortants in the next influenza season [57].

This manuscript presents the characterization of influenza strains collected from numerous surveillance programs and research studies, and as such has several limitations. As the collection and initial analysis of samples was conducted in numerous clinics and laboratories respectively, the influenza strains included in this study were subject to any sampling or testing biases that were inherent in the different studies. The analyses presented in the study were also limited to the data available to the NIC and did not include epidemiological information such as detailed admission data and rates of infection. In addition, samples were not collected throughout 2009–2011 for some of the studies (such as event-based surveillance for H1N1pdm09) and some of the study sites did not contribute samples throughout the entire study period. As a large number of samples were tested throughout the study (n = 10,813) only a representative number of isolates could be further analysed for genetic, antigenic and antiviral drug resistance characteristics. However, a large proportion of isolates were also sent to WHOCCs for further characterization (data not shown).

In summary, this study coupled with our previous study of influenza activity in Cambodia over a six year period from 2006 to 2011 provides strong evidence that influenza was prevalent in Cambodia and influenza activity occurred throughout the year with annual peaks during the rainy season from June to November. Since September 2009, there has been sustained circulation of predominantly A/H1N1pdm09 viruses. Seasonal influenza A/H3N2 and B virus have also circulated, although to a lesser extent. In 2010 there continued to be co-circulation of influenza A/H1N1pdm09, A/H3N2 and B viruses, with the latter two being predominant from November and December 2010 through 2011. The influenza virus epidemics in Cambodia occurred through genetically distinct multiple viruses, even if all of the isolates had similar antigenicity to the reference vaccine strain. The drug susceptibility profile of Cambodian influenza strains showed that neuraminidase inhibitors would be the drug of choice for influenza treatment and chemoprophylaxis in Cambodia, as adamantanes are no longer expected to be effective and not recommended for use [58]. Despite the reduced number of influenza vaccines used in Cambodia, policy makers should consider the use of a quadrivalent vaccine (when available) to counter the co-circulation of the two influenza A lineages.

## Supporting Information

Figure S1 **Monthly proportion of positive influenza samples among ILI specimens tested from 2009 to 2011 and among ALRI specimens tested from 2009 to July 2010.**
(TIFF)

Figure S2 **Amino acid alignment of HA1 domain of 28 representative A/H3N2 strains isolated from 2009 to 2011 in Cambodia with the vaccine strains A/Brisbane/10/2007 and A/Perth/16/2009.**
(RTF)

Figure S3 **Amino acid alignment of 33 HA sequences from A/H1N1pdm09 strains circulating from 2009 to 2011 in Cambodia with the vaccine strain A/California/07/2009.**
(RTF)

Figure S4 **Amino acid alignment of 20 HA1 sequences from influenza B strains circulating from 2009 to 2011 in Cambodia with the vaccine strain B/Malaysia/2506/2004 and B/Brisbane/60/2008.**
(RTF)

Table S1 **Outline of all of the surveillance programs and research studies that contributed to the present study.**
(DOCX)

Table S2 **All Cambodian influenza A/H3N2, A/H1N1pdm09 and Influenza B virus sequences included in the analysis are available via GISAID website (** www.gisaid.org).
(DOCX)

Table S3 **Resistance to adamantanes predicted following M gene sequencing of influenza A viruses, 2009–2011.**
(DOC)

Table S4 **Amino acid substitutions in A/H3N2 viruses isolated in Cambodia from 2009 to 2011.**
(DOCX)

Table S5 **Amino acid substitutions in A/H1N1pdm09 viruses isolated in Cambodia from 2009 to 2011.**
(DOCX)

Table S6 **Variations in HA1 amino acid sequences in influenza B-Victoria lineage viruses isolated in Cambodia in 2009, 2010 and 2011 by comparison with vaccine strain B/Malaysia/2506/2004.**
(DOCX)
