# Supplementary figures and images for: Epidemiological and Virological Characteristics of Influenza Viruses Circulating in Cambodia from 2009 to 2011
Source: PLoS One. 2014 Oct 23;9(10):e110713. doi: 10.1371/journal.pone.0110713 (PMC4207757; doi:10.1371/journal.pone.0110713)

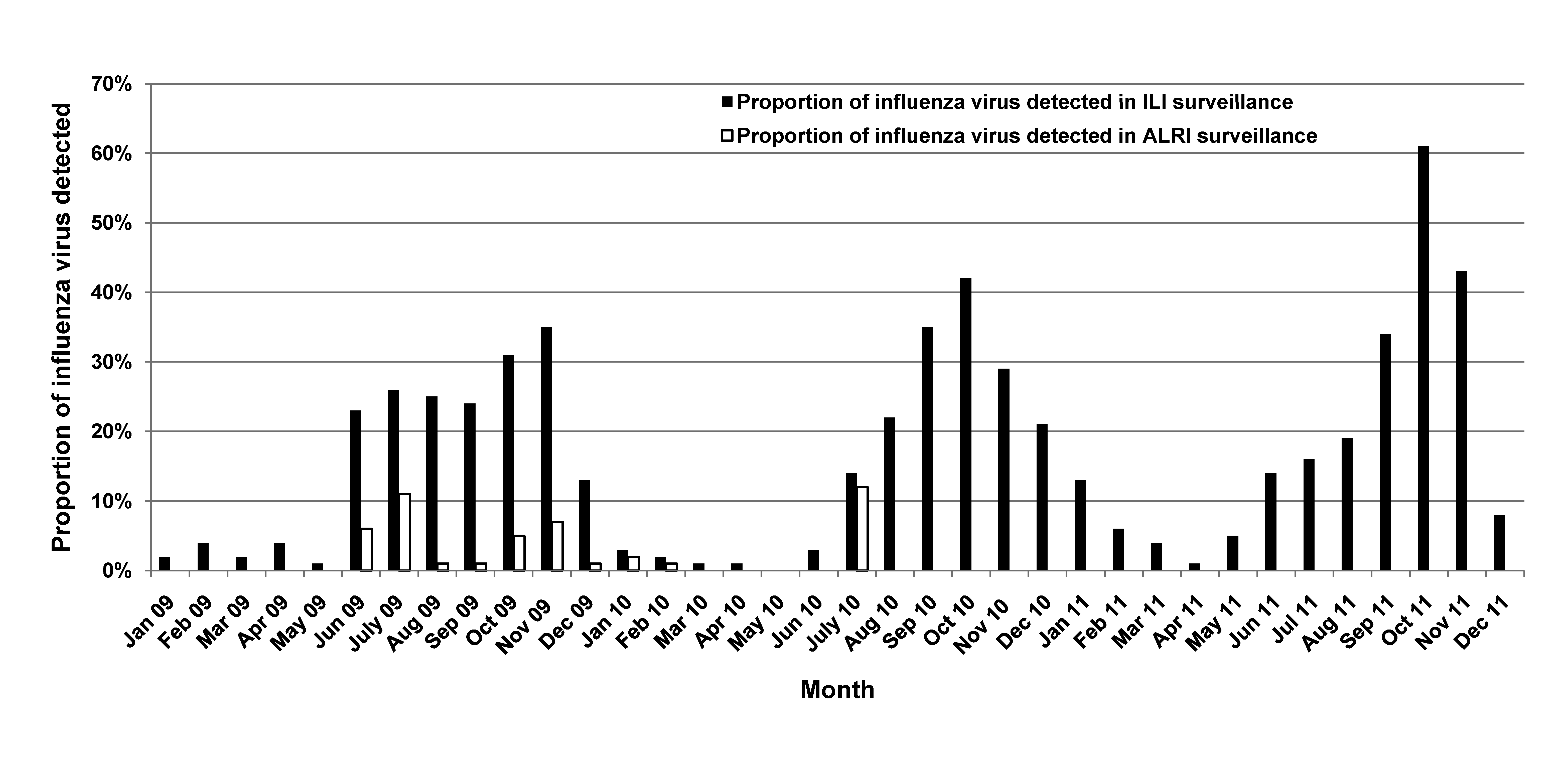

Supplement: Figure S1 — Monthly proportion of positive influenza samples among ILI specimens tested from 2009 to 2011 and among ALRI specimens tested from 2009 to July 2010. (TIFF) [file pone.0110713.s001.tiff]
