# Supplementary material for: Epidemiological and Virological Characteristics of Influenza Viruses Circulating in Cambodia from 2009 to 2011
Source: PLoS One. 2014 Oct 23;9(10):e110713. doi: 10.1371/journal.pone.0110713 (PMC4207757; doi:10.1371/journal.pone.0110713)
Supplement: Figure S2 — Amino acid alignment of HA1 domain of 28 representative A/H3N2 strains isolated from 2009 to 2011 in Cambodia with the vaccine strains A/Brisbane/10/2007 and A/Perth/16/2009. (RTF) [file pone.0110713.s002.rtf]

Figure S2. Amino acid alignment of HA1 domain of 28 representative A/H3N2 strains isolated from 2009 to 2011 in Cambodia with the vaccine strains A/Brisbane/10/2007 and A/Perth/16/2009. 

                                   10         20         30         40         50         60         70                   
                          ....|....| ....|....| ....|....| ....|....| ....|....| ....|....| ....|....| 
A/Brisbane/10/2007        QKLPGNDNST ATLCLGHHAV PNGTIVKTIT NDQIEVTNAT ELVQSSSTGE ICDSPHQILD GENCTLIDAL  70  
A/Cambodia/T092/2009      .......... .......... ....V..... .......... .......... .......... .K........  70  
A/Cambodia/T085/2009      .......... .......... ....V..... .......... .......... .......... .K........  70  
A/Cambodia/T103/2009      .......... .......... .......... .......... .......... .......... .K........  70  
A/Cambodia/T245/2009      .......... .......... .......... .......... .......... .......... .K........  70  
A/Cambodia/7/2009         .......... .......... .......... .......... .......... .......... .K........  70  
A/Cambodia/T140/2009      .......... .......... .......... .......... .......... .......... .K........  70  
A/Cambodia/T308/2009      .......... .......... .......... .......... .......... .......... .K........  70  
A/Cambodia/15/2009        .......... .......... .......... .......... .......... .......... .K........  70  
A/Cambodia/T284/2009      .......... .......... .......... .......... .......... .......... .K........  70  
A/Cambodia/T108/2009      .......... .......... .......... .......... .......... .......... .K........  70  
A/Cambodia/12/2009        .......... .......... .......... .......... .......... .......... .K........  70  
A/Cambodia/U0825342/2010  .......... .......... .......... .......... .......... .......... .K........  70  
A/Perth/16/2009e          .......... .......... .......... .......... .......... .......... .K........  70  
A/Cambodia/40/2011        .......... .......... .......... .......... ....N..I.. .......... ..........  70  
A/Cambodia/V1116321/2011  .......... .......... .......... .......... ....N..I.. .......... ..........  70  
A/Cambodia/V1013305/2011  .......... .......... .......... .......... ....N..I.. .......... ..........  70  
A/Cambodia/55/2011        .......... .......... .......... .......... ....N..I.. .......... ..........  70  
A/Cambodia/74/2011        .......... .......... .......... ..R....... ....N..I.. .......... ..........  70  
A/Cambodia/V1116330/2011  .......... .......... .......... ..R....... ....N..I.. .......... ..........  70  
A/Cambodia/V0902310/2011  .......... .......... .......... ..R....... ....N..I.. .......... ..........  70  
A/Cambodia/V1005380/2011  .......... .......... .......... ..R....... ....N..I.. .......... ..........  70  
A/Cambodia/39/2011        .......... .......... .......... ..R....... ....N..I.. .......... ..........  70  
A/Cambodia/U307/2010      .......... .......... .......... .......... .......... .......... ..........  70  
A/Cambodia/U371/2010      .......... .......... .......... .......... .......... ..N....... ..........  70  
A/Cambodia/U368/2010      .......... .......... .......... .......... .......... ..N....... ..........  70  
A/Cambodia/U5340/2010     .......... .......... .......... .......... .......... ..N....... ..........  70  
A/Cambodia/U424/2010      .......... .......... .......... .......... .......... ..N....... ..........  70  
A/Cambodia/U325/2010      .......... .......... .......... .......... .......... ..N....... ..........  70  
A/Cambodia/U349/2010      .......... .......... .......... .......... .......... ..N....... ..........  70  

                                   80         90        100        110        120        130        140              
                          ....|....| ....|....| ....|....| ....|....| ....|....| ....|....| ....|....| 
A/Brisbane/10/2007        LGDPQCDGFQ NKKWDLFVER SKAYSNCYPY DVPDYASLRS LVASSGTLEF NNESFNWTGV TQNGTSSACI  140 
A/Cambodia/T092/2009      .......... .......... .......... .......... .......... .......... ..........  140 
A/Cambodia/T085/2009      .......... .......... .......... .......... .......... .........L ..........  140 
A/Cambodia/T103/2009      .......... .......... .......... .......... .......... .......... ..........  140 
A/Cambodia/T245/2009      .......... .N........ ...H...... .......... .......M.. .......... ..........  140 
A/Cambodia/7/2009         .......... .......... ...H...... .......... .......... .......... ..........  140 
A/Cambodia/T140/2009      .......... .......... ...H...... .......... .......... .......... ..........  140 
A/Cambodia/T308/2009      .......... .T........ ...H...... .......... .......... .......... ..........  140 
A/Cambodia/15/2009        .......... .......... ...H...... .......... .......... .......... ..........  140 
A/Cambodia/T284/2009      .......... .......... ...H...... .......... .......... .......... ..........  140 
A/Cambodia/T108/2009      .......... .......... ...H...... .......... .......... .......... ..........  140 
A/Cambodia/12/2009        .......... .......... ...H...... .......... .......... .......... ..........  140 
A/Cambodia/U0825342/2010  .......... .......... ...H...... .......... .......... .......... ..........  140 
A/Perth/16/2009e          .......... .......... .......... .......... .......... .......... ..........  140 
A/Cambodia/40/2011        .......... .......... .......... .......... .I........ .......... .........M  140 
A/Cambodia/V1116321/2011  .......... .......... .......... .......... .......... .......... .........L  140 
A/Cambodia/V1013305/2011  .......... .......... .......... .......... .......... .......... .........M  140 
A/Cambodia/55/2011        .......... .......... .......... .......... .......... .......... .........M  140 
A/Cambodia/74/2011        .......... .......... .......... .......... .......... .......... ..........  140 
A/Cambodia/V1116330/2011  ...L...... .......... .......... .....T.... .......... .......... ..........  140 
A/Cambodia/V0902310/2011  .......... .......I.. .......... .......... .......... .......... ..........  140 
A/Cambodia/V1005380/2011  .......... .......... .......... .......... .......... .......... ..........  140 
A/Cambodia/39/2011        .......... .......... .......... .......... .......... .......... ..........  140 
A/Cambodia/U307/2010      .......... .......... ...H...... .......... .......... .......... ..........  140 
A/Cambodia/U371/2010      .......... .......... ...H...... .......... .......... .......... ..........  140 
A/Cambodia/U368/2010      .......... .......... ...H...... .......... .......... .......... ..........  140 
A/Cambodia/U5340/2010     .......... .......... ...H...... .......... .......... .......... ..........  140 
A/Cambodia/U424/2010      .......... .......... ...H...... .......... .......... .......... ..........  140 
A/Cambodia/U325/2010      .......... .......... ...H...... .......... .......... .......... ..........  140 
A/Cambodia/U349/2010      .......... .......... ...H...... .......... .......... .......... ..........  140 

                                  150        160        170        180        190        200        210            
                          ....|....| ....|....| ....|....| ....|....| ....|....| ....|....| ....|....| 
A/Brisbane/10/2007        RRSNNSFFSR LNWLTHLKFK YPALNVTMPN NEKFDKLYIW GVHHPGTDND QIFLYAQASG RITVSTKRSQ  210 
A/Cambodia/T092/2009      ...K...... .......N.. .......... ..Q....... ........K. .......... ..........  210 
A/Cambodia/T085/2009      ...K...... .......N.. .......... ..QS...... ........K. .......... ..........  210 
A/Cambodia/T103/2009      ...K...... .......N.. .......... ..Q....... ........K. .......... ..........  210 
A/Cambodia/T245/2009      ...K...... .......N.. .......... ..Q....... ........K. .......... ..........  210 
A/Cambodia/7/2009         ...K...... .......N.. .......... ..Q....... ........K. .......... ..........  210 
A/Cambodia/T140/2009      ...K...... .......N.. .......... ..Q....... ........K. .......... .T........  210 
A/Cambodia/T308/2009      ..YK...... .......N.. .......... ..Q....... ........K. .......... ..........  210 
A/Cambodia/15/2009        ...K...... .......N.. .......... ..Q....... ........K. .......... ..........  210 
A/Cambodia/T284/2009      ...K...... .......N.. .......... ..Q....... ........K. .......... ..........  210 
A/Cambodia/T108/2009      ...K...... .......N.. .......... ..Q....... ........K. .......... ..........  210 
A/Cambodia/12/2009        ...K...... .......N.. .......... ..Q....... ........K. .......... ..........  210 
A/Cambodia/U0825342/2010  ...K...... .......N.. .......... ..Q....... ........K. .......... ..........  210 
A/Perth/16/2009e          ...K...... .......N.. .......... ..Q....... ..L.....K. .......... ..........  210 
A/Cambodia/40/2011        ....S..... .......N.. .......... ..Q....... ........K. .......S.. ..........  210 
A/Cambodia/V1116321/2011  ...SC..... .......N.. .......... ..Q....... ........K. .......S.. ..........  210 
A/Cambodia/V1013305/2011  ....S..... .......N.. .......... ..Q....... ........K. .......S.. ..........  210 
A/Cambodia/55/2011        ....S..... .......N.. .......... ..Q....... ........K. .......S.. ..........  210 
A/Cambodia/74/2011        .......... .......N.. .......... ..Q....... ........K. .......S.. ..........  210 
A/Cambodia/V1116330/2011  .......... .......N.. .......... ..Q....... ........K. .......S.. ..........  210 
A/Cambodia/V0902310/2011  .......... .......N.. .......... ..Q....... ........K. .......S.. ..........  210 
A/Cambodia/V1005380/2011  .......... .......N.. .......... ..Q....... ........K. .......S.. ..........  210 
A/Cambodia/39/2011        .......... .......N.. .......... ..Q....... ........K. .......S.. ..........  210 
A/Cambodia/U307/2010      .......... .......N.. .......... ..Q....... ........K. .......... ..........  210 
A/Cambodia/U371/2010      .......... .......N.. .......... ..Q....... ........K. .......... .......K..  210 
A/Cambodia/U368/2010      .......... .......N.. .......... ..Q....... ........K. .......... .......K..  210 
A/Cambodia/U5340/2010     .......... .......N.. .......... ..Q....... ........K. .......... .......K..  210 
A/Cambodia/U424/2010      .......... .......N.. .......... ..Q....... ........K. .......... ..........  210 
A/Cambodia/U325/2010      .......... .......N.. .......... ..Q....... ........K. .......... ..........  210 
A/Cambodia/U349/2010      .......... .......N.. .......... ..Q....... ........K. .......... ..........  210 

                                  220        230        240        250        260        270        280            
                          ....|....| ....|....| ....|....| ....|....| ....|....| ....|....| ....|....| 
A/Brisbane/10/2007        QTVIPNIGSR PRVRNIPSRI SIYWTIVKPG DILLINSTGN LIAPRGYFKI RSGKSSIMRS DAPIGKCNSE  280 
A/Cambodia/T092/2009      .......... .......... .......... .......... .......... .......... ..........  280 
A/Cambodia/T085/2009      .......... .......... .......... .......... .......... .......... ..........  280 
A/Cambodia/T103/2009      .......... .......... .......... .......... .......... .......... ..........  280 
A/Cambodia/T245/2009      .......... .......... .......... .......... .......... .......... ..........  280 
A/Cambodia/7/2009         .......... ..I....... .......... .......... .......... .......... ..........  280 
A/Cambodia/T140/2009      .......... ..I....... .......... .L........ .......... .......... ..........  280 
A/Cambodia/T308/2009      .......... .......... .......... .L........ .......... .......... ..........  280 
A/Cambodia/15/2009        .......... .......... .......... .L........ .......... .......... ..........  280 
A/Cambodia/T284/2009      .......... .......... .......... .L........ .......... .......... ..........  280 
A/Cambodia/T108/2009      .......... .......... .......... .L........ .......... .......... ..........  280 
A/Cambodia/12/2009        .......... .......... .......... .L........ .......... .......... ..........  280 
A/Cambodia/U0825342/2010  .......... .......... .......... .L........ .......... .......... ..........  280 
A/Perth/16/2009e          ...S...... .......... .......... .......... .......... .......... ..........  280 
A/Cambodia/40/2011        .A........ ..I....... .......... .......... .......... .......... ..........  280 
A/Cambodia/V1116321/2011  .A........ ..I....... .......... .......... .......... .......... ..........  280 
A/Cambodia/V1013305/2011  .A........ ..I....... .......... .......... .......... .......... ..........  280 
A/Cambodia/55/2011        .A........ ..I....... .......... .......... .......... .......... ..........  280 
A/Cambodia/74/2011        .A........ T.I....... .......... .......... .......... .......... .......K..  280 
A/Cambodia/V1116330/2011  .A........ L.I....... .......... .......... .......... .......... .......K..  280 
A/Cambodia/V0902310/2011  .A........ ..I.D..... .......... .......... .......... .......... .......K..  280 
A/Cambodia/V1005380/2011  .A........ ..I....... .......... .......... .......... .......... .......K..  280 
A/Cambodia/39/2011        .A........ ..I....... .......... .......... .......... .......... .......K..  280 
A/Cambodia/U307/2010      .A........ .........V .......... .......... .......... .......... .........A  280 
A/Cambodia/U371/2010      .A........ .........V .......... .......... .......... .......... .........A  280 
A/Cambodia/U368/2010      .A........ .........V .......... .......... .......... .......... .........A  280 
A/Cambodia/U5340/2010     .A........ .........V .......... .......... .......... .......... .........A  280 
A/Cambodia/U424/2010      .A........ .........V .......... .......... .......... .......... .........A  280 
A/Cambodia/U325/2010      .A........ .........V .......... .......... .......... .......... .........A  280 
A/Cambodia/U349/2010      .A........ L........V .......... .......... .......... .......... .........A  280 

                                  290        300        310        320              
                          ....|....| ....|....| ....|....| ....|....| ....|....
A/Brisbane/10/2007        CITPNGSIPN DKPFQNVNRI TYGACPRYVK QNTLKLATGM RNVPEKQTR 329 
A/Cambodia/T092/2009      .......... .......... .......... .......... ......... 329 
A/Cambodia/T085/2009      .......... .......... .......... .......... ......... 329 
A/Cambodia/T103/2009      .......... .......... .......... .......... ......... 329 
A/Cambodia/T245/2009      .......... .......... .......... .......... ......... 329 
A/Cambodia/7/2009         .......... .......... .......... .......... ......... 329 
A/Cambodia/T140/2009      .......... .......... .......... .......... ........K 329 
A/Cambodia/T308/2009      .......... .......... .......... .......... ........K 329 
A/Cambodia/15/2009        .......... .......... .......... .......... ........K 329 
A/Cambodia/T284/2009      .......... .......... .......... .......... ........K 329 
A/Cambodia/T108/2009      .......... .......... .......... .......... ........K 329 
A/Cambodia/12/2009        .......... .......... .......... .......... ........K 329 
A/Cambodia/U0825342/2010  .......... .......... .......... .......... ........K 329 
A/Perth/16/2009e          .......... .......... .......... .......... ......... 329 
A/Cambodia/40/2011        .......... .......... ........I. .S........ ......... 329 
A/Cambodia/V1116321/2011  .......... .......... .......... .S........ ......... 329 
A/Cambodia/V1013305/2011  .......... .......... .......... .S........ ......... 329 
A/Cambodia/55/2011        .......... .......... .......... .S........ ......... 329 
A/Cambodia/74/2011        .......... .......... .......... .S........ ......... 329 
A/Cambodia/V1116330/2011  .......... .......... .......... .S........ ......... 329 
A/Cambodia/V0902310/2011  .......... .......... .......... .S........ ......... 329 
A/Cambodia/V1005380/2011  .......... .......... .......... .S........ ......... 329 
A/Cambodia/39/2011        .......... .......... .......... .S........ ......... 329 
A/Cambodia/U307/2010      ........T. .......... .......... .......... ......... 329 
A/Cambodia/U371/2010      .......... .......... .......... .......... ......... 329 
A/Cambodia/U368/2010      .......... .......... .......... .......... ......... 329 
A/Cambodia/U5340/2010     .......... .......... .......... .......... ......... 329 
A/Cambodia/U424/2010      ......C... .......... .......... .......... ......... 329 
A/Cambodia/U325/2010      .......... .......... .......... .......... ......... 329 
A/Cambodia/U349/2010      .......... .......... .......... .......... ......... 329 


Identity to reference vaccine strain A/Brisbane/10/2007 is indicated by a dot. 
