# Supplementary material for: Epidemiological and Virological Characteristics of Influenza Viruses Circulating in Cambodia from 2009 to 2011
Source: PLoS One. 2014 Oct 23;9(10):e110713. doi: 10.1371/journal.pone.0110713 (PMC4207757; doi:10.1371/journal.pone.0110713)
Supplement: Figure S3 — Amino acid alignment of 33 HA sequences from A/H1N1pdm09 strains circulating from 2009 to 2011 in Cambodia with the vaccine strain A/California/07/2009. (RTF) [file pone.0110713.s003.rtf]

Figure S3. Amino acid alignment of 33 HA sequences from A/H1N1pdm09 strains circulating from 2009 to 2011 in Cambodia with the vaccine strain A/California/07/2009. 
                                   10         20         30         40         50         60         70                   
                          ....|....| ....|....| ....|....| ....|....| ....|....| ....|....| ....|....| 
A/California//07/2009e    DTLCIGYHAN NSTDTVDTVL EKNVTVTHSV NLLEDKHNGK LCKLRGVAPL HLGKCNIAGW ILGNPECESL  70  
A/Cambodia/T021/2009      .......... .......... .......... .......... .......... .......... ..........  70  
A/Cambodia/T320/2009      .......... .......... .......... .......... .......... .......... ..........  70  
A/Cambodia/T354/2009      .......... .......... .......... .......... .......... .......... ..........  70  
A/Cambodia/T093/2009      .......... .......... .......... .......... .......... .......... ..........  70  
A/Cambodia/U094/2010      .......... .......... .......... ......N... .......... .......... ..........  70  
A/Cambodia/T217/2009      .......... .......... .......... .......... .......... .......... ..........  70  
A/Cambodia/T282/2009      .......... .......... .......... .......... .......... .......... ..........  70  
A/Cambodia/T272/2009      .......... .......... .......... .......... .......... .......... ..........  70  
A/Cambodia/T057/2009      .......... .......... .......... .......... .......... .......... ..........  70  
A/Cambodia/U301/2010      .......... .......... .......... .......... .......... .......... ..........  70  
A/Cambodia/T234/2009      .......... .......... .......... .......... .......... .......... ..........  70  
A/Cambodia/T068/2009      .......... .......... .......... .......... .......... .......... ..........  70  
A/Cambodia/T075/2009      .......... .......... .......... .......... .......... .......... ..........  70  
A/Cambodia/T028/2009      ....V..... .......... .......... .......... .......... .......... ..........  70  
A/Cambodia/U219/2010      .......... .......... .......... .......... .......... .......... ..........  70  
A/Cambodia/U306/2010      .......... .......... .......... .......... .......... .......... ..........  70  
A/Cambodia/U326/2010      .......... .......... .......... .......... .......... .......... ..........  70  
A/Cambodia/U099/2010      .......... .......... .......... .......... .......... .......... ..........  70  
A/Cambodia/9/2010         .......... .......... .......... .......... .......... .......... ..........  70  
A/Cambodia/8/2010         .......... .......... .......... .......... .......... .......... ..........  70  
A/Cambodia/10/2010        .......... .......... .......... .......... .......... .......... ..........  70  
A/Cambodia/72/2010        .......... .......... .......... .......... .......... .......... ..........  70  
A/Cambodia/V0608350/2011  .......... .......... .......... .......... .......... .......... ..........  70  
A/Cambodia/V0721310/2011  .......... .......... .......... .......... .......... .......... ..........  70  
A/Cambodia/15/2011        .......... .......... .......... .......... .......... .......... ..........  70  
A/Cambodia/69/2011        .......... .......... .......... .......... .......... .......... ..........  70  
A/Cambodia/70/2011        .......... ........I. .......... .......... .......... .......... ..........  70  
A/Cambodia/V0601312/2011  .......... .......... .......... .......... .......... .......... ..........  70  
A/Cambodia/V0902314/2011  .......... .......... .......... .......... .......... .......... .........F  70  
A/Cambodia/V1005346/2011  .......... .......... .......... .......... .......... .......... ..........  70  
A/Cambodia/V1019320/2011  .......... .......... .......... .......... .......... .......... ..........  70  
A/Cambodia/59/2011        .......... .......... .......... .......... .......... .......... ..........  70  
A/Cambodia/53/2011        .......... .......... .......... .......... .......... .......... ..........  70  

                                   80         90        100        110        120        130        140              
                          ....|....| ....|....| ....|....| ....|....| ....|....| ....|....| ....|....| 
A/California//07/2009e    STASSWSYIV ETPSSDNGTC YPGDFIDYEE LREQLSSVSS FERFEIFPKT SSWPNHDSNK GVTAACPHAG  140 
A/Cambodia/T021/2009      .......... ..S....... .......... .......... .......... .......... ..........  140 
A/Cambodia/T320/2009      .......... ..S....... .......... .......... .......... .......... ..........  140 
A/Cambodia/T354/2009      .......... ..S....... .......... .......... .......... .......... ..........  140 
A/Cambodia/T093/2009      .......... ..S..G.... .......... .......... .......... .......... ..........  140 
A/Cambodia/U094/2010      .......... ..S....... .......... .......... .....M.... .......... ..........  140 
A/Cambodia/T217/2009      .......... ..S....... .......... .......... .......... .......... ..........  140 
A/Cambodia/T282/2009      .......... ..S....... .......... .......... .......... .......... ..........  140 
A/Cambodia/T272/2009      .......... ..S....... .......... .......... .......... .......... ..........  140 
A/Cambodia/T057/2009      .......... ..S....... .......... .......... .......... .......... ..........  140 
A/Cambodia/U301/2010      A......... ..SN...... .......... .......... .......... .......... ..........  140 
A/Cambodia/T234/2009      .......... ..S....... .......... .......... .......... .......... ..........  140 
A/Cambodia/T068/2009      .......... ..S....... .......... .......... .......... .......... ..........  140 
A/Cambodia/T075/2009      .......... ..S....... .......... .......... .......... .......... ..........  140 
A/Cambodia/T028/2009      .......... ..S....... .......... .......... .......... .......... ..........  140 
A/Cambodia/U219/2010      .......... ..S....... .......... .......... .......... .......... ..........  140 
A/Cambodia/U306/2010      .......... ..S....... .......... .......... .......... .......... ..........  140 
A/Cambodia/U326/2010      .......... ..S....... .......... .......... .......... ....D..... ..........  140 
A/Cambodia/U099/2010      .......... ..S....... .......... .......... .......... ....D..... ..........  140 
A/Cambodia/9/2010         .......... ..S....... .......... .......... .......... .......... ..........  140 
A/Cambodia/8/2010         .......... ..S....... .......... .......... .......... .......... ..........  140 
A/Cambodia/10/2010        .......... ..S....... .......... .......... .......... .......... ..........  140 
A/Cambodia/72/2010        .......... ..S....... .......... .......... .......... .......... ..........  140 
A/Cambodia/V0608350/2011  .......... ..S....... .......... .......... .......... ....D..... ..........  140 
A/Cambodia/V0721310/2011  .......... ..S....... .......... .......... .......... .......... ..........  140 
A/Cambodia/15/2011        .......... ..S....... .......... .......... .......... .......... ..........  140 
A/Cambodia/69/2011        .......... ..S....... .......... .......... .......... .......... ..........  140 
A/Cambodia/70/2011        .......... ..S....... .......... .......... .......... .......... ..........  140 
A/Cambodia/V0601312/2011  .......... ..S....... .......... .......... .......... .......... ..........  140 
A/Cambodia/V0902314/2011  .......... ..S....... .......... .......... .......... .......... ..........  140 
A/Cambodia/V1005346/2011  .......... ..S....... .......... .......... .......... .......... ..........  140 
A/Cambodia/V1019320/2011  .......... ..S....... .......... .....N.... .......... .......... ..........  140 
A/Cambodia/59/2011        .......... ..S....... .......... .......... .......... .......... ..........  140 
A/Cambodia/53/2011        .......... ..S....... ......N... .......... .......... N......... ..........  140 

                                  150        160        170        180        190        200        210            
                          ....|....| ....|....| ....|....| ....|....| ....|....| ....|....| ....|....| 
A/California//07/2009e    AKSFYKNLIW LVKKGNSYPK LSKSYINDKG KEVLVLWGIH HPSTSADQQS LYQNADAYVF VGSSRYSKKF  210 
A/Cambodia/T021/2009      .......... .......... .......... .......... .......... .......... ..........  210 
A/Cambodia/T320/2009      .......... .......... .......... .......... .......... .......... ..T.......  210 
A/Cambodia/T354/2009      .......... .......... .......... .......... .......... .......... ..T.......  210 
A/Cambodia/T093/2009      .......... .......... .......... .......... .......... .......... ..T.......  210 
A/Cambodia/U094/2010      .......... .......... .......... .......... .......... .......... ..T.......  210 
A/Cambodia/T217/2009      .......... .......... .......... .......... .......... .......... ..T.......  210 
A/Cambodia/T282/2009      .......... .......... .......... .......... .......... .......... ..T.K.....  210 
A/Cambodia/T272/2009      .......... .......... .......... .......... .......... .......... ..T.......  210 
A/Cambodia/T057/2009      .......... .......... .......... .......... .......... .......... ..T.K.....  210 
A/Cambodia/U301/2010      .......... .......... .......... R......... .......... ......T... ..T.......  210 
A/Cambodia/T234/2009      .......... .......... .......... .......... .......... .......... ..T.......  210 
A/Cambodia/T068/2009      .......... .......... .......... .......... .......... .......... ..T.......  210 
A/Cambodia/T075/2009      .......... .......... .......... .......... .......... .......... ..T.......  210 
A/Cambodia/T028/2009      .......... .......... .......... .......... .......... .......... ..T.......  210 
A/Cambodia/U219/2010      .......... .......... .......... .......... .......... .......... ..T.......  210 
A/Cambodia/U306/2010      .......... .......... .......... .......... .......... .......... ..T.......  210 
A/Cambodia/U326/2010      .......... .......... .......... .......... .......... .......... ..T.......  210 
A/Cambodia/U099/2010      .......... .......... .......... .......... .......... .......... ..T.......  210 
A/Cambodia/9/2010         .......... .......... .......... .......... .......... .......... ..T.......  210 
A/Cambodia/8/2010         .......... .......... .......... .......... .......... .......... ..T.......  210 
A/Cambodia/10/2010        .......... .......... .......... .......... .......... .......... ..T.......  210 
A/Cambodia/72/2010        .......... .......... .......... .......... ....T..... ......T... ..T.......  210 
A/Cambodia/V0608350/2011  ..G....... .......... .......... .......... ....T..... ......T... ..T.......  210 
A/Cambodia/V0721310/2011  ..G....... .....D.... .......... .......... ...NT..... ......T... ..T.......  210 
A/Cambodia/15/2011        ..G....... .......... .......... .......... ....I..... ......X... ..T.......  210 
A/Cambodia/69/2011        ..G....... .........T .......... .......... ....T..... ......T... ..T.......  210 
A/Cambodia/70/2011        ..G....... .......... .......... .......... ....T..... ......T... ..T.......  210 
A/Cambodia/V0601312/2011  ..G....... .......... .......... .......... ....T..... ......T... .WT.......  210 
A/Cambodia/V0902314/2011  ..G.....V. .......... .......... .......... ..P.T..... ......T... ..T.......  210 
A/Cambodia/V1005346/2011  ..G....... .......... .......... .......... ....T..... ......T... ..T.......  210 
A/Cambodia/V1019320/2011  ..G....... .......... .......... .......... ....T..... ......T... .ET.......  210 
A/Cambodia/59/2011        ..G....... .......... .......... .......... ....T..... ......T... ..T.......  210 
A/Cambodia/53/2011        ..G....... .......... .......... .......... ....T..... ......T... ..T.......  210 

                                  220        230        240        250        260        270        280            
                          ....|....| ....|....| ....|....| ....|....| ....|....| ....|....| ....|....| 
A/California//07/2009e    KPEIAIRPKV RDREGRMNYY WTLVEPGDKI TFEATGNLVV PRYAFAMERN AGSGIIISDT PVHDCNTTCQ  280 
A/Cambodia/T021/2009      .......... ..Q....... .......... .......... .......... .......... ..........  280 
A/Cambodia/T320/2009      .......... ..Q....... .......... .......... .......... .......... ..........  280 
A/Cambodia/T354/2009      .......... ..Q....... .......... .......... .......... .......... ..........  280 
A/Cambodia/T093/2009      .......... ..Q....... .......... .......... .......... .......... ..........  280 
A/Cambodia/U094/2010      .......... ..Q....... .......... .......... .......... .......... ..........  280 
A/Cambodia/T217/2009      .......... ..Q....... .......... .......... .........D .......... ..........  280 
A/Cambodia/T282/2009      .......... ..Q....... .......... .......... .......... .......... ..........  280 
A/Cambodia/T272/2009      .......... ..Q....... .......... .......... .......... .......... ..........  280 
A/Cambodia/T057/2009      .......... ..Q....... .......... .......... .......... .......... ..........  280 
A/Cambodia/U301/2010      .......... ..Q....... .......... .......... .......... .......... ..........  280 
A/Cambodia/T234/2009      .......... ..Q....... .......... .......... .......... .......... ..........  280 
A/Cambodia/T068/2009      .......... ..Q....... .......... .......... .......... .......... ..........  280 
A/Cambodia/T075/2009      .......... ..Q....... .......... .......... .......... .......... ..........  280 
A/Cambodia/T028/2009      .......... ..Q....... .......... .......... .......... .......... ..........  280 
A/Cambodia/U219/2010      .......... ..Q....... .......... .......... .......... .......... ..........  280 
A/Cambodia/U306/2010      .......... ..Q....... .......... .......... .......... .......... ..........  280 
A/Cambodia/U326/2010      .......... ..Q....... .......... .......... .......... .......... ..........  280 
A/Cambodia/U099/2010      .......... ..Q....... .......... .......... .......... .......... ..........  280 
A/Cambodia/9/2010         .......... ..Q....... .......... .......... .......... .......... ..........  280 
A/Cambodia/8/2010         .......... ..Q....... .......... .......... .......... .......... ..........  280 
A/Cambodia/10/2010        .......... ..Q....... .......... .......... .......... .......... ..........  280 
A/Cambodia/72/2010        .......... ..Q....... .......... .......... .......... .......... ..........  280 
A/Cambodia/V0608350/2011  .......... ..Q....... .......... .......... .......... .......... ..........  280 
A/Cambodia/V0721310/2011  .......... ..Q....... .......... .......... .........G .......... ..........  280 
A/Cambodia/15/2011        .......... ..Q...V... .......... .......... .........D .......... ..........  280 
A/Cambodia/69/2011        .......... ..Q....... .......... .......... .........D .......... ..........  280 
A/Cambodia/70/2011        .......... ..Q....... .......... .......... .........D .......... ..........  280 
A/Cambodia/V0601312/2011  .......... ..Q....... .......... .......... .........D .......... ..........  280 
A/Cambodia/V0902314/2011  .......... ..Q....... .......... .......... .........D .......... ..........  280 
A/Cambodia/V1005346/2011  ....T..... ..Q....... .......... .......... .........D .......... ..........  280 
A/Cambodia/V1019320/2011  .......... ..Q....... .......... .......... .........D .......... ..........  280 
A/Cambodia/59/2011        .......... ..Q....... .......... .......... .........D .......... ..........  280 
A/Cambodia/53/2011        .......... ..Q....... .......... .......... .........D .......... ..........  280 

                                  290        300        310        320        330        340        350            
                          ....|....| ....|....| ....|....| ....|....| ....|....| ....|....| ....|....| 
A/California//07/2009e    TPKGAINTSL PFQNIHPITI GKCPKYVKST KLRLATGLRN IPSIQSRGLF GAIAGFIEGG WTGMVDGWYG  350 
A/Cambodia/T021/2009      .......... ..H....... .......... .......... V......... .......... ..........  350 
A/Cambodia/T320/2009      .......... .......... .......... .......... V......... .......... ..........  350 
A/Cambodia/T354/2009      .......... .......... .......... .......... V......... .......... ..........  350 
A/Cambodia/T093/2009      .......... .......... .......... .......... V......... .......... ..........  350 
A/Cambodia/U094/2010      .......... .......... .......... .......... V......... .......... ..........  350 
A/Cambodia/T217/2009      .......... .......... .......... .......... V......... .......... ..........  350 
A/Cambodia/T282/2009      .......... .......... .......... .......... V......... .......... ..........  350 
A/Cambodia/T272/2009      .......... .......... .......... .......... V......... .......... ..........  350 
A/Cambodia/T057/2009      .......... .......... .......... .......... V......... .......... ..........  350 
A/Cambodia/U301/2010      .......... .......... .......... .......... V......... .......... ..........  350 
A/Cambodia/T234/2009      .......... .......... .......... .......... V......... .......... ..........  350 
A/Cambodia/T068/2009      .......... .......... .......... .......... V......... .......... ..........  350 
A/Cambodia/T075/2009      .......... .......... .......... .......... V......... .......... ..........  350 
A/Cambodia/T028/2009      .......... .......... .......... .......... V......... .......... ..........  350 
A/Cambodia/U219/2010      .......... .......... .......... .......... V......... .......... ..........  350 
A/Cambodia/U306/2010      .......... .......... .......... .......... V......... .......... ..........  350 
A/Cambodia/U326/2010      .......... .......... .......... .......... V......... .......... ..........  350 
A/Cambodia/U099/2010      .......... .......... .......... .......... V......... .......... ..........  350 
A/Cambodia/9/2010         .......... .......... .......... .......... V......... .......... ..........  350 
A/Cambodia/8/2010         .......... .......... .......... .......... V......... .......... ..........  350 
A/Cambodia/10/2010        .......... .......... .......... .......... V......... .......... ..........  350 
A/Cambodia/72/2010        .......... .......... .......... .......... V......... .......... ..........  350 
A/Cambodia/V0608350/2011  .......... .......... .......... .......... V......... .......... ..........  350 
A/Cambodia/V0721310/2011  .......... .......... .......... .......... V.....K... .......... ..........  350 
A/Cambodia/15/2011        .......... .......... .......... .......... V......... .......... ..........  350 
A/Cambodia/69/2011        .......... .......... .......... .......... V......... .......... ..........  350 
A/Cambodia/70/2011        .......... .......... .......... .......... V......... .......... ..........  350 
A/Cambodia/V0601312/2011  .......... .......... .......... .......... V......... .......... ..........  350 
A/Cambodia/V0902314/2011  .......... .......... .......... .......... V......... .......... ..........  350 
A/Cambodia/V1005346/2011  .......... .......... .......... .......... V......... .......... ..........  350 
A/Cambodia/V1019320/2011  .......... ..H....... .......... .......... V......... .......... ..........  350 
A/Cambodia/59/2011        .......... .......... .......... .......... V......... .......... ..........  350 
A/Cambodia/53/2011        .......... .......... .......... .......... V......... .......... ..........  350 

                                  360        370        380        390        400        410        420            
                          ....|....| ....|....| ....|....| ....|....| ....|....| ....|....| ....|....| 
A/California//07/2009e    YHHQNEQGSG YAADLKSTQN AIDEITNKVN SVIEKMNTQF TAVGKEFNHL EKRIENLNKK VDDGFLDIWT  420 
A/Cambodia/T021/2009      .......... .......... .......... .......... .......... .......... ..........  420 
A/Cambodia/T320/2009      .......... .......... .......... .......... .......... .......... ..........  420 
A/Cambodia/T354/2009      .......... .......... .......... .......... .......... .......... ..........  420 
A/Cambodia/T093/2009      .......... .......... .......... .......... .......... .......... ..........  420 
A/Cambodia/U094/2010      .......... .......... .......... .......... .......... .......... ..........  420 
A/Cambodia/T217/2009      .......... .......... .......... .......... .......... .......... I.........  420 
A/Cambodia/T282/2009      .......... .......... .......... .......... .......... .......... I.........  420 
A/Cambodia/T272/2009      .......... .......... .......... .......... .......... .......... I.........  420 
A/Cambodia/T057/2009      .......... .......... .......... .......... .......... .......... ..........  420 
A/Cambodia/U301/2010      .......... .......... .......... .......... .......... .......... ..........  420 
A/Cambodia/T234/2009      .......... .......... .......... .......... .......... .......... ..........  420 
A/Cambodia/T068/2009      .......... .......... .......... .......... .......... .......... ..........  420 
A/Cambodia/T075/2009      .......... .......... .......... .......... .......... .......... ..........  420 
A/Cambodia/T028/2009      .......... .......... .......... .......... .......... .......... ..........  420 
A/Cambodia/U219/2010      .......... .......... ...K...... .......... .......... .......... ..........  420 
A/Cambodia/U306/2010      .......... .......... ...K...... .......... .......... .......... ..........  420 
A/Cambodia/U326/2010      .......... .......... ...K...... .......... .......... .......... ..........  420 
A/Cambodia/U099/2010      .......... .......... ...K...... .......... .......... .......... ..........  420 
A/Cambodia/9/2010         .......... .......... ...K...... .......... .......... .......... ..........  420 
A/Cambodia/8/2010         .......... .......... ...K...... .......... .......... .......... ..........  420 
A/Cambodia/10/2010        .......... .......... ...K...... .......... .......... .......... ..........  420 
A/Cambodia/72/2010        .......... .......... ...K...... .......... .......... .......... ..........  420 
A/Cambodia/V0608350/2011  .......... .......... ...K...... .......... .......... .......... ..........  420 
A/Cambodia/V0721310/2011  .......... .......... ...K...... .......... .......... .......... ..........  420 
A/Cambodia/15/2011        .......... .......... ...K...... .......... .......... .......... ..........  420 
A/Cambodia/69/2011        .......... .......... ...K...... .......... .......... .......... ..........  420 
A/Cambodia/70/2011        .......... .......... ...K...... .......... .......... .......... ..........  420 
A/Cambodia/V0601312/2011  .......... .......... ...K...... .......... .......... .......... ..........  420 
A/Cambodia/V0902314/2011  .......... .......... ...K...... .......... .......... .......... ..........  420 
A/Cambodia/V1005346/2011  .......... .......... ...K...... .......... .......... .......... ..........  420 
A/Cambodia/V1019320/2011  .......... .......... ...K...... .......... .......... .......... ..........  420 
A/Cambodia/59/2011        .......... .......... ...K...... .......... .......... .......... ..........  420 
A/Cambodia/53/2011        .......... .......... ...K...... .......... .......... .......... ..........  420 

                                  430        440        450        460        470        480        490            
                          ....|....| ....|....| ....|....| ....|....| ....|....| ....|....| ....|....| 
A/California//07/2009e    YNAELLVLLE NERTLDYHDS NVKNLYEKVR SQLKNNAKEI GNGCFEFYHK CDNTCMESVK NGTYDYPKYS  490 
A/Cambodia/T021/2009      ..T....... .......... .......... .......... .......... .......... ..........  490 
A/Cambodia/T320/2009      .......... .......... .......... .......... .......... .......... ..........  490 
A/Cambodia/T354/2009      .......... .......... .......... .......... .......... .......... ..........  490 
A/Cambodia/T093/2009      .......... .......... .......... .......... .......... .......... ..........  490 
A/Cambodia/U094/2010      .......... .......... .......... .........V .......... .......... ..........  490 
A/Cambodia/T217/2009      .......... .......... .......... .......... .......... .......... ..........  490 
A/Cambodia/T282/2009      .......... .......... .......... .......... .......... .......... ..........  490 
A/Cambodia/T272/2009      .......... .......... .......... .......... .......... .......... ..........  490 
A/Cambodia/T057/2009      .......... .......... .......... .......... .......... .......... ..........  490 
A/Cambodia/U301/2010      .......... .......... .......... .......... .......... .......... ..........  490 
A/Cambodia/T234/2009      .......... .......... .......... .......... .......... .......... ..........  490 
A/Cambodia/T068/2009      .......... .......... .......... .......... .......... .......... ..........  490 
A/Cambodia/T075/2009      .......... .......... .......... .......... .......... .......... ..........  490 
A/Cambodia/T028/2009      .......... .......... .......... .......... .......... .......... ..........  490 
A/Cambodia/U219/2010      .......... .......... .......... .......... .......... .......... ..........  490 
A/Cambodia/U306/2010      .......... .......... .......... .......... .......... .......... ..........  490 
A/Cambodia/U326/2010      .......... .......... .......... .......... .......... .......... ..........  490 
A/Cambodia/U099/2010      .......... .......... .......... .......... .......... .......... ..........  490 
A/Cambodia/9/2010         .......... .......... .......... N......... .......... .......... ..........  490 
A/Cambodia/8/2010         .......... .......... .......... .......... .......... .......... ..........  490 
A/Cambodia/10/2010        .......... .......... .......... .......... .......... .......... ..........  490 
A/Cambodia/72/2010        .......... .......... .L........ N......... .......... .......... ..........  490 
A/Cambodia/V0608350/2011  .......... .......... .......... N......... .......... .......... ..........  490 
A/Cambodia/V0721310/2011  .......... .......... .......... N......... .......... .......... ..........  490 
A/Cambodia/15/2011        .......... .......... .......... N......... .......... .......... ..........  490 
A/Cambodia/69/2011        .......... .......... .......... N......... .......... .....T.... ..........  490 
A/Cambodia/70/2011        .......... .......... .......... N......... .......... ...K...... ..........  490 
A/Cambodia/V0601312/2011  .......... .......... .......... N......... .......... .......... ..........  490 
A/Cambodia/V0902314/2011  .......... .......... .......... N..E...... .......... ......K... ..........  490 
A/Cambodia/V1005346/2011  .......... .......... .......... N......... .......... .....I.... ..........  490 
A/Cambodia/V1019320/2011  .......... .......... .......... N......... .......... .......... ..........  490 
A/Cambodia/59/2011        .......... .......... .......... N......... .......... .......... ..........  490 
A/Cambodia/53/2011        .......... .......... .......... N......... .......... .......... ..........  490 

                                  500        510        520        530        540          
                          ....|....| ....|....| ....|....| ....|....| ....|....| ....
A/California//07/2009e    EEAKLNREEI DGVKLESTRI YQILAIYSTV ASSLVLVVSL GAISFWMCSN GSLQ 544 
A/Cambodia/T021/2009      .......... .......... .......... .......... .......... .... 544 
A/Cambodia/T320/2009      .......... .......... .......... .......... .......... .... 544 
A/Cambodia/T354/2009      .......... .......... .......... .......... .......... .... 544 
A/Cambodia/T093/2009      .......... .......... .......... .......... .......... .... 544 
A/Cambodia/U094/2010      .......... .......... .......... .......... .......... .... 544 
A/Cambodia/T217/2009      .......... .......... .......... .......... .......... .... 544 
A/Cambodia/T282/2009      .......... .......... .......... .......... .......... .... 544 
A/Cambodia/T272/2009      .......... .......... .......... .......... .......... .... 544 
A/Cambodia/T057/2009      .......... .......... .......... .......... .......... .... 544 
A/Cambodia/U301/2010      .......... .......... .......... .......... .......... .... 544 
A/Cambodia/T234/2009      .......... .......... .......... .......... .......... .... 544 
A/Cambodia/T068/2009      .......... .......... .......... .......... .......... .... 544 
A/Cambodia/T075/2009      .......... .......... .......... .......... .......... .... 544 
A/Cambodia/T028/2009      .......... .......... .......... .......... .......... .... 544 
A/Cambodia/U219/2010      .......... .......... .......... .......... .......... .... 544 
A/Cambodia/U306/2010      .......... N......... .......... .......... .......... .... 544 
A/Cambodia/U326/2010      .......... .......... .......... .......... .......... .... 544 
A/Cambodia/U099/2010      .......... .......... .......... .......... .......... .... 544 
A/Cambodia/9/2010         .......... .......... .......... .......... .......... .... 544 
A/Cambodia/8/2010         .......... .......... .......... .......... .......... .... 544 
A/Cambodia/10/2010        .......... .......... .......... .......... .......... .... 544 
A/Cambodia/72/2010        .......... .......... .......... .......... .......... ...  543 
A/Cambodia/V0608350/2011  .......... .......... .......... .......... .......... .... 544 
A/Cambodia/V0721310/2011  ........K. .......... .......... .......... .......... .... 544 
A/Cambodia/15/2011        ........K. .......... .......... .......... .......... .... 544 
A/Cambodia/69/2011        ........K. .......... .......... .......... .......... .... 544 
A/Cambodia/70/2011        ........K. .......... .......... .......... .......... .... 544 
A/Cambodia/V0601312/2011  ........K. .......... .......... .......... .......... .... 544 
A/Cambodia/V0902314/2011  ........K. .......... .......... .......... .......... .... 544 
A/Cambodia/V1005346/2011  ........K. .......... .......... .......... .......... .... 544 
A/Cambodia/V1019320/2011  ........K. .......... .......... .......... .......... .... 544 
A/Cambodia/59/2011        ........K. .......... .......... .......... .......... .... 544 
A/Cambodia/53/2011        ........K. .......... .......... .......... .......... .... 544 


Identity to reference vaccine strain A/California/07/2009 is indicated by a dot. 
