# Supplementary material for: Epidemiological and Virological Characteristics of Influenza Viruses Circulating in Cambodia from 2009 to 2011
Source: PLoS One. 2014 Oct 23;9(10):e110713. doi: 10.1371/journal.pone.0110713 (PMC4207757; doi:10.1371/journal.pone.0110713)
Supplement: Figure S4 — Amino acid alignment of 20 HA1 sequences from influenza B strains circulating from 2009 to 2011 in Cambodia with the vaccine strain B/Malaysia/2506/2004 and B/Brisbane/60/2008. (RTF) [file pone.0110713.s004.rtf]

Figure S4. Amino acid alignment of 20 HA1 sequences from influenza B strains circulating from 2009 to 2011 in Cambodia with the vaccine strain B/Malaysia/2506/2004 and B/Brisbane/60/2008. 
                                   10         20         30         40         50         60         70                   
                          ....|....| ....|....| ....|....| ....|....| ....|....| ....|....| ....|....| 
B/Malaysia/2506/2004      DRICTGITSS NSPHVVKTAT QGEVNVTGVI PLTTTPTKSH FANLKGTETR GKLCPKCLNC TDLDVALGRP  70  
B/Cambodia/V0112324/2011  .......... .......... .......... ......I... .......... .......... ..........  70  
B/Cambodia/30/2011        .......... .......... .......... S.....I... .......... .......... ..........  70  
B/Cambodia/V0316324/2011  .......... .......... .......... ......I... .......... .......... ..........  70  
B/Cambodia/U1103348/2010  .......... .......... .......... ......I... .......... .......... ..........  70  
B/Cambodia/V0629331/2011  .......... .......... .......... ......I... .......... .......... ..........  70  
B/Cambodia/V0602318/2011  .......... .......... .......... ......I... .......... .......... ..........  70  
B/Brisbane/60/2008        .......... .......... .......... .......... .......... .......... ..........  70  
B/Cambodia/T1231151/2009  ......V... .......... .......... .......... .......... .....R.... ..........  70  
B/Cambodia/T0910051/2009  .......... .......... .......... .......... .......... .....R.... ..........  70  
B/Cambodia/T1119143/2009  .......... .......... .......... .......... .......... .....R.... ..........  70  
B/Cambodia/U1102389/2010  .......... .......... .......... .......... .......... .....R.... ..........  70  
B/Cambodia/U0707330/2010  .......... .......... .......... .......... .......... .....R.... ..........  70  
B/Cambodia/V1221355/2011  .......... .......... .......... .......... .......... .......... ..........  70  
B/Cambodia/106/2011       .......... .......... .......... .......... .......... .......... ..........  70  
B/Cambodia/V0825326/2011  .......... .......... .......... .......... .......... .......... ..........  70  
B/Cambodia/V1103320/2011  .......... .......... .......... .......... .......... .......... ..........  70  
B/Cambodia/V1012330/2011  .......... .......... .......... .......... .......... .......... ..........  70  
B/Cambodia/V0825316/2011  .......... .......... .......... .......... .......... .......... ..........  70  
B/Cambodia/V0112308/2011  .......... .......... .......... .......... .......... .......P.. ..........  70  
B/Cambodia/U1013320/2010  .......... .......... .......... .......... .......... .......P.. ..........  70  
B/Cambodia/U1006332/2010  .......... .......... .......... .......... .......... .......P.. ..........  70  

                                   80         90        100        110        120        130        140              
                          ....|....| ....|....| ....|....| ....|....| ....|....| ....|....| ....|....| 
B/Malaysia/2506/2004      KCTGNIPSAR VSILHEVRPV TSGCFPIMHD RTKIRQLPNL LRGYEHIRLS THNVINAENA PGGSYKIGTS  140 
B/Cambodia/V0112324/2011  .......... .......... .......... .......... .......... .......... ...P......  140 
B/Cambodia/30/2011        .......... .......... .......... .......... .......... .......... ...P......  140 
B/Cambodia/V0316324/2011  .......... .........I .......... .......... .......... .......... ...P......  140 
B/Cambodia/U1103348/2010  .......... .......... .......... .......... .......... .......... ...P......  140 
B/Cambodia/V0629331/2011  .......... .......... .......... .......... .......... .......... ...P......  140 
B/Cambodia/V0602318/2011  .......... .......... .......... .......... .......... .......... ...P......  140 
B/Brisbane/60/2008        ....K..... .......... .......... .......... .......... .......... ...P......  140 
B/Cambodia/T1231151/2009  ....K..... .......... .......... .......... .......... .......... ...P......  140 
B/Cambodia/T0910051/2009  ....K..... .......... .......... .......... .......... .......... ...P......  140 
B/Cambodia/T1119143/2009  ....K..... .......... .......... .......... .......... .......... ...P......  140 
B/Cambodia/U1102389/2010  ....K..... .......... .......... .......... .......... .......... ...P......  140 
B/Cambodia/U0707330/2010  ....K..... .......... .......... .......... .......... .......... ...P......  140 
B/Cambodia/V1221355/2011  ....K..... .......... .......... .......... .......... ........D. ...P......  140 
B/Cambodia/106/2011       ....K..... .......... .......... .......... .......... ........D. ...P......  140 
B/Cambodia/V0825326/2011  ....K..... .......... .......... .......... .......... ........D. ...P......  140 
B/Cambodia/V1103320/2011  ....K..... .......... .......... .......... .......... .......... ...P......  140 
B/Cambodia/V1012330/2011  ....K..... .......... .......... .......... .......... .......... ...P......  140 
B/Cambodia/V0825316/2011  ....K..... .......... .......... .......... .......... .......... ...P......  140 
B/Cambodia/V0112308/2011  ....K..... .......... .......... .......... .......... .......... ...P......  140 
B/Cambodia/U1013320/2010  ....K..... .......... .......... .......... .......... .......... ...P......  140 
B/Cambodia/U1006332/2010  ....K..... .......... .......... .......... .......... .......... ...P......  140 

                                  150        160        170        180        190        200        210            
                          ....|....| ....|....| ....|....| ....|....| ....|....| ....|....| ....|....| 
B/Malaysia/2506/2004      GSCPNVTNGN GFFATMAWAV PKNDNNKTAT NSLTIEVPYI CTEGEDQITV WGFHSDNEAQ MAKLYGDSKP  210 
B/Cambodia/V0112324/2011  .......... .......... .......... .......... .......... ........T. ..........  210 
B/Cambodia/30/2011        .......... .......... .......... .......... .......... ......S.T. ..........  210 
B/Cambodia/V0316324/2011  .......... .......... .......... .......... .......... ........T. ..........  210 
B/Cambodia/U1103348/2010  .......... .......... .......... .......... .......... ........T. ..........  210 
B/Cambodia/V0629331/2011  .......... .......... .......... .......... .......... ........T. ..........  210 
B/Cambodia/V0602318/2011  .......... .......... .......... .......... .......... ........T. ..........  210 
B/Brisbane/60/2008        .....I.... .......... ....K..... .P........ .......... ........T. ..........  210 
B/Cambodia/T1231151/2009  .....I.... .......... ....K..... .P........ .......... ........T. ..........  210 
B/Cambodia/T0910051/2009  .....I.... .......... ....K..... .P........ .......... ........T. ..........  210 
B/Cambodia/T1119143/2009  .....I.... .......... ....K..... .P........ .......... ........T. ..........  210 
B/Cambodia/U1102389/2010  .....I.... .......... ....K..... .P........ .......... ........T. ..........  210 
B/Cambodia/U0707330/2010  .....I.... .......... .N..K..... .P........ .......... ........T. ..........  210 
B/Cambodia/V1221355/2011  .......... .......... ....K..... .P........ .......... ........T. ..........  210 
B/Cambodia/106/2011       .......... .......... ....K..... .P........ .......... ........T. ..........  210 
B/Cambodia/V0825326/2011  .......... .......... ....K..... .P........ .......... ........T. ..........  210 
B/Cambodia/V1103320/2011  .......... ...E...... ....K..... .P........ .......... ........T. ..........  210 
B/Cambodia/V1012330/2011  .......... ...E...... ....K..... .P........ .......... ........T. ..........  210 
B/Cambodia/V0825316/2011  .......... ...E...... ....K..... .P........ .......... ........T. ..........  210 
B/Cambodia/V0112308/2011  .......... .......... ....K..... .P........ .......... ........T. ..........  210 
B/Cambodia/U1013320/2010  .......... .......... ....K..... .P........ .......... ........T. ..........  210 
B/Cambodia/U1006332/2010  .......... .......... ....K..... .P........ .......... ........T. ..........  210 

                                  220        230        240        250        260        270        280            
                          ....|....| ....|....| ....|....| ....|....| ....|....| ....|....| ....|....| 
B/Malaysia/2506/2004      QKFTSSANGV TTHYVSQIGG FPNQTEDGGL PQSGRIVVDY MVQKSGKTGT ITYQRGILLP QKVWCASGRS  280 
B/Cambodia/V0112324/2011  .......... .......... .......... .......... .......... .......... ..........  280 
B/Cambodia/30/2011        .......... .......... .......... .......... .......... .......... ..........  280 
B/Cambodia/V0316324/2011  .......... .......... .......... .......... .......... .......... ..........  280 
B/Cambodia/U1103348/2010  .......... .......... .......... .......... .......... .......... ..........  280 
B/Cambodia/V0629331/2011  .......... .......... .......... .......... .......... .......... ..........  280 
B/Cambodia/V0602318/2011  .......... .......... .......... .......... .......... .......... ..........  280 
B/Brisbane/60/2008        .......... .......... .......... .......... .......... .......... ..........  280 
B/Cambodia/T1231151/2009  .......... .......... .......... .......... .......... .......... ..........  280 
B/Cambodia/T0910051/2009  .......... .......... .......... .......... .......... .......... ..........  280 
B/Cambodia/T1119143/2009  .......... .......... .......... .......... .......... .......... ..........  280 
B/Cambodia/U1102389/2010  .......... .......... .......... .......... .......... .......... ..........  280 
B/Cambodia/U0707330/2010  .......... .......... .......... .......... .......... .......... ..........  280 
B/Cambodia/V1221355/2011  .......... .......... .......... .......... .......... .......... ..........  280 
B/Cambodia/106/2011       .......... .......... .......... .......... .......... .......... ..........  280 
B/Cambodia/V0825326/2011  .......... .......... .......... .......... .......... .......... ..........  280 
B/Cambodia/V1103320/2011  .......... .......... .......... .......... ....P..... .......... ..........  280 
B/Cambodia/V1012330/2011  .......... .......... .......... .......... .......... .......... ..........  280 
B/Cambodia/V0825316/2011  .......... .......... .......... .......... .......... .......... ..........  280 
B/Cambodia/V0112308/2011  .......... .......... .......... .......... .......... .......... ..........  280 
B/Cambodia/U1013320/2010  .......... .......... .......... .......... .......... .......... ..........  280 
B/Cambodia/U1006332/2010  .......... .......... .......... .......... .......... .......... ..........  280 

                                  290        300        310        320        330        340        350            
                          ....|....| ....|....| ....|....| ....|....| ....|....| ....|....| ....|....| 
B/Malaysia/2506/2004      KVIKGSLPLI GEADCLHEKY GGLNKSKPYY TGEHAKAIGN CPIWVKTPLK LANGTKYRPP AKLLKERGFF  350 
B/Cambodia/V0112324/2011  .......... .......... .......... .......... .........R .......... ..........  350 
B/Cambodia/30/2011        .......... .......... .......... .......... .......... .......... ..........  350 
B/Cambodia/V0316324/2011  .......... .......... .......... .......... .......... .......... ..........  350 
B/Cambodia/U1103348/2010  .......... .......... .......... .......... .......... .......... ..........  350 
B/Cambodia/V0629331/2011  .......... .......... .......... .......... .......... .......... ..........  350 
B/Cambodia/V0602318/2011  .......... .......... .......... .......... .......... .......... ..........  350 
B/Brisbane/60/2008        .......... .......... .......... .......... .......... .......... ..........  350 
B/Cambodia/T1231151/2009  .......... .......... .......... .......... .......... .......... ..........  350 
B/Cambodia/T0910051/2009  .......... .......... .......... .......... .......... .......... ..........  350 
B/Cambodia/T1119143/2009  .......... .......... .......... .......... .......... .......... ..........  350 
B/Cambodia/U1102389/2010  .......... .......... .......... .......... .......... .......... ....R.....  350 
B/Cambodia/U0707330/2010  .......... .......... .......... .......... .......... .......... ..........  350 
B/Cambodia/V1221355/2011  .......... .......... .......... .......... .......... .......... ..........  350 
B/Cambodia/106/2011       .......... .......... .......... .......... .......... .......... ..........  350 
B/Cambodia/V0825326/2011  .......... .......... .......... .......... .......... .......... ..........  350 
B/Cambodia/V1103320/2011  .......... .......... .......... .......... .......... .......... ..........  350 
B/Cambodia/V1012330/2011  .......... .......... .......... .......... .......... .......... ..........  350 
B/Cambodia/V0825316/2011  .......... .......... .......... .......... .......... .......... ..........  350 
B/Cambodia/V0112308/2011  .......... .......... .......... .......... .......... .......... ..........  350 
B/Cambodia/U1013320/2010  .......... .......... .......... .......... .......... .......... ..........  350 
B/Cambodia/U1006332/2010  .......... .......... .......... .......... .......... .......... ..........  350 

                                  360        370        380        390        400        410        420            
                          ....|....| ....|....| ....|....| ....|....| ....|....| ....|....| ....|....| 
B/Malaysia/2506/2004      GAIAGFLEGG WEGMIAGWHG YTSHGAHGVA VAADLKSTQE AINKITKNLN SLSELEVKNL QRLSGAMDEL  420 
B/Cambodia/V0112324/2011  .......... .......... .......... .......... .......... .......... ..........  420 
B/Cambodia/30/2011        .......... .......... .......... .......... .......... .......... ..........  420 
B/Cambodia/V0316324/2011  .......... .......... .......... .......... .......... .......... ..........  420 
B/Cambodia/U1103348/2010  .......... .......... .......... .......... .......... .......... ..........  420 
B/Cambodia/V0629331/2011  .......... .......... .......... .......... .......... .......... ..........  420 
B/Cambodia/V0602318/2011  .......... .......... .......... .......... ....V..... .......... ..........  420 
B/Brisbane/60/2008        .......... .......... .......... .......... .......... .......... ..........  420 
B/Cambodia/T1231151/2009  .......... .......... .......... .......... .......... .......... ..........  420 
B/Cambodia/T0910051/2009  .......... .......... .......... .......... .......... .......... ..........  420 
B/Cambodia/T1119143/2009  .......... .......... .......... .......... .......... .......... ..........  420 
B/Cambodia/U1102389/2010  .......... .......... .......... .......... .......... .......... ..........  420 
B/Cambodia/U0707330/2010  .......... .......... .......... .......... .......... .......... ..........  420 
B/Cambodia/V1221355/2011  .......... .......... .......... .......... .......... .......... ..........  420 
B/Cambodia/106/2011       .......... .......... .......... .......... .......... .......... ..........  420 
B/Cambodia/V0825326/2011  .......... .......... .......... .......... .......... .......... ..........  420 
B/Cambodia/V1103320/2011  .......... .......... .......... .......... .......... .......... ..........  420 
B/Cambodia/V1012330/2011  .......... .......... .......... .......... .......... .......... ..........  420 
B/Cambodia/V0825316/2011  .......... .......... .......... .......... .......... .......... ..........  420 
B/Cambodia/V0112308/2011  .......... .......... .......... .......... .......... .......... ..........  420 
B/Cambodia/U1013320/2010  .......... .......... .......... .......... .......... .......... ..........  420 
B/Cambodia/U1006332/2010  .......... .......... .......... .......... .......... .......... ..........  420 

                                  430        440        450        460        470        480        490            
                          ....|....| ....|....| ....|....| ....|....| ....|....| ....|....| ....|....| 
B/Malaysia/2506/2004      HNEILELDEK VDDLRADTIS SQIELAVLLS NEGIINSEDE HLLALERKLK KMLGPSAVEI GNGCFETKHK  490 
B/Cambodia/V0112324/2011  .......... .......... .......... .......... .......... .......... ..........  490 
B/Cambodia/30/2011        .......... .......... .......... .......... .......... .......... ..........  490 
B/Cambodia/V0316324/2011  .......... .......... .......... .......... .......... .......... ..........  490 
B/Cambodia/U1103348/2010  .......... .......... .......... .......... .......... .......... ..........  490 
B/Cambodia/V0629331/2011  .......... .......... .......... .......... .......... .......... ..........  490 
B/Cambodia/V0602318/2011  .......... .......... .......... .......... .......... .......... ..........  490 
B/Brisbane/60/2008        .......... .......... .......... .......... .......... .......... ..........  490 
B/Cambodia/T1231151/2009  .......... .......... .......... .......... .......... .......... ..........  490 
B/Cambodia/T0910051/2009  .......... .......... .......... .......... .......... .......... ..........  490 
B/Cambodia/T1119143/2009  .......... .......... .......... .......... .......... .......... ..........  490 
B/Cambodia/U1102389/2010  .......... .......... .......... .......... .......... .......... ..........  490 
B/Cambodia/U0707330/2010  .......... .......... .......... .......... .......... .......... ..........  490 
B/Cambodia/V1221355/2011  .......... .......... .......... .......... .......... .......... ..........  490 
B/Cambodia/106/2011       .......... .......... .......... .......... .......... .......... ..........  490 
B/Cambodia/V0825326/2011  .......... .......... .......... .......... .......... .......... ..........  490 
B/Cambodia/V1103320/2011  .......... .......... .......... .......... .......... .......... ..........  490 
B/Cambodia/V1012330/2011  .......... .......... .......... .......... .......... .......... ..........  490 
B/Cambodia/V0825316/2011  .......... .......... .......... .......... .......... .......... ..........  490 
B/Cambodia/V0112308/2011  .......... .......... .......... .......... .......... .......... ..........  490 
B/Cambodia/U1013320/2010  .......... .......... .......... .......... .......... .......... ..........  490 
B/Cambodia/U1006332/2010  .......... .......... .......... .......... .......... .......... ..........  490 

                                  500        510        520        530        540        550        560            
                          ....|....| ....|....| ....|....| ....|....| ....|....| ....|....| ....|....| 
B/Malaysia/2506/2004      CNQTCLDRIA AGTFDAGEFS LPTFDSLNIT AASLNDDGLD NHTILLYYST AASSLAVTLM IAIFVVYMVS  560 
B/Cambodia/V0112324/2011  .......... .......... .......... .......... .......... .......... ..........  560 
B/Cambodia/30/2011        .......... .......... .......... .......... .......... .......... ..........  560 
B/Cambodia/V0316324/2011  .......... .......... .......... .......... .......... .......... ..........  560 
B/Cambodia/U1103348/2010  .......... .......... .......... .......... .......... .......... ..........  560 
B/Cambodia/V0629331/2011  .......... .......... .......... .......... .......... .......... ..........  560 
B/Cambodia/V0602318/2011  .......... .......... .......... .......... .......... .......... ..........  560 
B/Brisbane/60/2008        .......... .......... .......... .......... .......... .......... ..........  560 
B/Cambodia/T1231151/2009  .......... .......... .......... .......... .......... .......... ....I.....  560 
B/Cambodia/T0910051/2009  .......... .......... .......... .......... .......... .......... ..........  560 
B/Cambodia/T1119143/2009  .......... .......... .......... .......... .......... .......... ..........  560 
B/Cambodia/U1102389/2010  .......... .......... .......... .......... .......... .......... ..........  560 
B/Cambodia/U0707330/2010  .......... .......... .......... .......... .......... .......... ..........  560 
B/Cambodia/V1221355/2011  .......... .......... .......... .......... .......... .......... ........I.  560 
B/Cambodia/106/2011       .......... .......... .......... .......... .......... .......... ........I.  560 
B/Cambodia/V0825326/2011  .......... .......... .......... .......... .......... .......... ........I.  560 
B/Cambodia/V1103320/2011  .......... .......... .......... .......... .......... .......... ........I.  560 
B/Cambodia/V1012330/2011  .......... .......... .......... .......... .......... .......... ........I.  560 
B/Cambodia/V0825316/2011  .......... .......... .........N .......... .......... .......... ........I.  560 
B/Cambodia/V0112308/2011  .......... .......... .......... .......... .......... .......... ..........  560 
B/Cambodia/U1013320/2010  .......... .......... .......... .......... .......... .......... ..........  560 
B/Cambodia/U1006332/2010  .......... .......... .......... .......... .......... .......... ..........  560 

                                  570 
                          ....|....| .
B/Malaysia/2506/2004      RDNVSCSICL * 571 
B/Cambodia/V0112324/2011  .......... * 571 
B/Cambodia/30/2011        .......... * 571 
B/Cambodia/V0316324/2011  .......... * 571 
B/Cambodia/U1103348/2010  .......... * 571 
B/Cambodia/V0629331/2011  .......... * 571 
B/Cambodia/V0602318/2011  .......... * 571 
B/Brisbane/60/2008        .......... * 571 
B/Cambodia/T1231151/2009  .......... * 571 
B/Cambodia/T0910051/2009  .......... * 571 
B/Cambodia/T1119143/2009  .......... * 571 
B/Cambodia/U1102389/2010  .......... * 571 
B/Cambodia/U0707330/2010  .......... * 571 
B/Cambodia/V1221355/2011  .......... * 571 
B/Cambodia/106/2011       .......... * 571 
B/Cambodia/V0825326/2011  .......... * 571 
B/Cambodia/V1103320/2011  .......... * 571 
B/Cambodia/V1012330/2011  .......... * 571 
B/Cambodia/V0825316/2011  .......... * 571 
B/Cambodia/V0112308/2011  .......... * 571 
B/Cambodia/U1013320/2010  .......... * 571 
B/Cambodia/U1006332/2010  .......... * 571 

Identity to reference vaccine strain B/Malaysia/2506/2004 is indicated by a dot. 
