# Supplementary material for: Epidemiological and Virological Characteristics of Influenza Viruses Circulating in Cambodia from 2009 to 2011
Source: PLoS One. 2014 Oct 23;9(10):e110713. doi: 10.1371/journal.pone.0110713 (PMC4207757; doi:10.1371/journal.pone.0110713)
Supplement: Table S1 — Outline of all of the surveillance programs and research studies that contributed to the present study. (DOCX) [file pone.0110713.s005.docx]

**Table S1. Outline of all of the surveillance programs and research studies that contributed to the present study.**

| **Type of study/surveillance** | **Period of study** | **Inclusion criteria** | **Mean age of positive patients** | **Number of samples positive (%)** | | |
| --- | --- | --- | --- | --- | --- | --- |
|  |  |  |  | **H1N1pdm09** | **H3N2** | **Influenza B** |
| ILI sentinel surveillance | January 2009 -December 2011 | An ILI case was defined by the sudden onset of fever (≥38°C axillary temperature) and cough or sore throat in the absence of other diagnosis | 7.3 | 26.6 | 32.9 | 40.5 |
| Event-based surveillance for pandemic A/H1N1 2009 | May 2009 -December 2010 | Inclusion criteria included any person with acute febrile respiratory illness (fever ≥38^o^C and respiratory symptoms, e.g. cough, sore throat, difficulty breathing) with no other apparent diagnosis and one or more of the following exposures to the risk of A/H1N1pdm09 virus infection within 7 days prior to symptoms onset: a) Close contact with a probable or confirmed case of A/H1N1pdm09 virus infection; b) Residing in or travelled to a province or foreign country with confirmed community transmission of A/H1N1pdm09 virus; c) Is part of a cluster of ILI cases; d) Handled specimens suspected of containing A/H1N1pdm09 virus. | 24 | 93 | 7 | N/A |
| ALRI study | January 2009 - July 2010 | For the ALRI study, in children under 5, a suspect case was defined as an illness of <10 days duration with cough or breathing difficulties plus tachypnea. For the 5-14 years age group, case definition included the above symptoms plus fever (≥38°C axillary temperature) on admission. For patients over 15 years old, a case was defined as a person with fever (≥38°C axillary temperature) on admission plus tachypnea or chest pain or auscultatory crackles. | 16.9 | 27 | 56 | 17 |

N/A: Not Applicable
