# Supplementary material for: Epidemiological and Virological Characteristics of Influenza Viruses Circulating in Cambodia from 2009 to 2011
Source: PLoS One. 2014 Oct 23;9(10):e110713. doi: 10.1371/journal.pone.0110713 (PMC4207757; doi:10.1371/journal.pone.0110713)
Supplement: Table S2 — All Cambodian influenza A/H3N2, A/H1N1pdm09 and Influenza B virus sequences included in the analysis are available via GISAID website ( www.gisaid.org). (DOCX) [file pone.0110713.s006.docx]

**Table S2. All Cambodian influenza A/H3N2, A/H1N1pdm09 and Influenza B virus sequences included in the analysis are available via GISAID website** ([www.gisaid.org](http://www.gisaid.org)).

| **GISAID Accession#** | **Segment** | **Country** | **Collection date** | **Isolate name** |
| --- | --- | --- | --- | --- |
| EPI341417 | HA | Cambodia | 2009-Sep-01 | A/Cambodia/T085/2009 |
| EPI341416 | HA | Cambodia | 2009-Jul-14 | A/Cambodia/T092/2009 |
| EPI341415 | HA | Cambodia | 2009-May-27 | A/Cambodia/T103/2009 |
| EPI341414 | HA | Cambodia | 2009-Jul-12 | A/Cambodia/T108/2009 |
| EPI341413 | HA | Cambodia | 2009-Nov-25 | A/Cambodia/T140/2009 |
| EPI341410 | HA | Cambodia | 2009-Jun-22 | A/Cambodia/T245/2009 |
| EPI341409 | HA | Cambodia | 2009-Jul-23 | A/Cambodia/T284/2009 |
| EPI341408 | HA | Cambodia | 2009-Jul-20 | A/Cambodia/T308/2009 |
| EPI341406 | HA | Cambodia | 2010-Aug-02 | A/Cambodia/U307/2010 |
| EPI341405 | HA | Cambodia | 2010-Aug-04 | A/Cambodia/U325/2010 |
| EPI341404 | HA | Cambodia | 2010-Sep-14 | A/Cambodia/U349/2010 |
| EPI341403 | HA | Cambodia | 2010-Sep-10 | A/Cambodia/U368/2010 |
| EPI341402 | HA | Cambodia | 2010-Sep-09 | A/Cambodia/U371/2010 |
| EPI341399 | HA | Cambodia | 2010-Aug-18 | A/Cambodia/U424/2010 |
| EPI341397 | HA | Cambodia | 2010-Aug-19 | A/Cambodia/U5340/2010 |
| EPI447005 | HA | Cambodia | 2011-Aug-24 | A/Cambodia/V0902310/2011 |
| EPI447018 | HA | Cambodia | 2011-Nov-06 | A/Cambodia/V1116330/2011 |
| EPI447017 | HA | Cambodia | 2011-Nov-15 | A/Cambodia/V1116321/2011 |
| EPI447016 | HA | Cambodia | 2011-Oct-10 | A/Cambodia/V1013305/2011 |
| EPI447015 | HA | Cambodia | 2011-Oct-03 | A/Cambodia/V1005380/2011 |
| EPI370296 | HA | Cambodia | 2011-Nov-21 | A/CAMBODIA/39/2011 |
| EPI370045 | HA | Cambodia | 2011-Nov-22 | A/CAMBODIA/40/2011 |
| EPI228268 | HA | Cambodia | 2009-Jun-24 | A/CAMBODIA/7/2009 |
| EPI228226 | HA | Cambodia | 2009-Jun-24 | A/CAMBODIA/12/2009 |
| EPI210287 | HA | Cambodia | 2009-Jun-16 | A/CAMBODIA/15/2009 |
| EPI370318 | HA | Cambodia | 2011-Nov-17 | A/CAMBODIA/74/2011 |
| EPI370321 | HA | Cambodia | 2011-Dec-05 | A/CAMBODIA/55/2011 |
| EPI464420 | HA | Cambodia | 2010-Aug-20 | A/Cambodia/U0825342/2010 |
| EPI341396 | HA | Cambodia | 2009-Jul-15 | A/Cambodia/T021/2009 |
| EPI341395 | HA | Cambodia | 2009-Jun-22 | A/Cambodia/T028/2009 |
| EPI341394 | HA | Cambodia | 2009-Aug-01 | A/Cambodia/T057/2009 |
| EPI341393 | HA | Cambodia | 2009-Sep-05 | A/Cambodia/T068/2009 |
| EPI341392 | HA | Cambodia | 2009-Jun-25 | A/Cambodia/T075/2009 |
| EPI341391 | HA | Cambodia | 2009-Jul-07 | A/Cambodia/T093/2009 |
| EPI341387 | HA | Cambodia | 2009-Aug-30 | A/Cambodia/T217/2009 |
| EPI341386 | HA | Cambodia | 2009-Nov-10 | A/Cambodia/T234/2009 |
| EPI341385 | HA | Cambodia | 2009-Jun-25 | A/Cambodia/T272/2009 |
| EPI341384 | HA | Cambodia | 2009-Jul-02 | A/Cambodia/T282/2009 |
| EPI341383 | HA | Cambodia | 2009-Sep-10 | A/Cambodia/T320/2009 |
| EPI341382 | HA | Cambodia | 2009-Sep-11 | A/Cambodia/T354/2009 |
| EPI341380 | HA | Cambodia | 2010-Jan-04 | A/Cambodia/U094/2010 |
| EPI341566 | HA | Cambodia | 2010-Mar-15 | A/Cambodia/U099/2010 |
| EPI341376 | HA | Cambodia | 2010-Feb-11 | A/Cambodia/U219/2010 |
| EPI341373 | HA | Cambodia | 2010-Jun-27 | A/Cambodia/U301/2010 |
| EPI341371 | HA | Cambodia | 2010-Jul-16 | A/Cambodia/U306/2010 |
| EPI341564 | HA | Cambodia | 2010-Jul-15 | A/Cambodia/U326/2010 |
| EPI447024 | HA | Cambodia | 2011-Oct-12 | A/Cambodia/V1019320/2011 |
| EPI447023 | HA | Cambodia | 2011-Sep-22 | A/Cambodia/V1005346/2011 |
| EPI447022 | HA | Cambodia | 2011-Aug-24 | A/Cambodia/V0902314/2011 |
| EPI447021 | HA | Cambodia | 2011-Jul-18 | A/Cambodia/V0721310/2011 |
| EPI447020 | HA | Cambodia | 2011-Jun-01 | A/Cambodia/V0608350/2011 |
| EPI447019 | HA | Cambodia | 2011-May-17 | A/Cambodia/V0601312/2011 |
| EPI357639 | HA | Cambodia | 2011-Jul-26 | A/CAMBODIA/15/2011 |
| EPI331408 | HA | Cambodia | 2010-Nov-18 | A/CAMBODIA/72/2010 |
| EPI279150 | HA | Cambodia | 2010-Jul-16 | A/CAMBODIA/10/2010 |
| EPI279147 | HA | Cambodia | 2010-Jul-14 | A/CAMBODIA/9/2010 |
| EPI279144 | HA | Cambodia | 2010-Jul-14 | A/CAMBODIA/8/2010 |
| EPI370230 | HA | Cambodia | 2011-Nov-04 | A/CAMBODIA/70/2011 |
| EPI370236 | HA | Cambodia | 2011-Nov-03 | A/CAMBODIA/69/2011 |
| EPI370251 | HA | Cambodia | 2011-Nov-08 | A/CAMBODIA/53/2011 |
| EPI376851 | HA | Cambodia | 2011-Sep-20 | A/CAMBODIA/59/2011 |
| EPI447004 | HA | Cambodia | 2011-Dec-17 | B/Cambodia/V1221355/2011 |
| EPI447002 | HA | Cambodia | 2011-Oct-26 | B/Cambodia/V1103320/2011 |
| EPI447001 | HA | Cambodia | 2011-Oct-10 | B/Cambodia/V1012330/2011 |
| EPI446998 | HA | Cambodia | 2011-Aug-17 | B/Cambodia/V0825326/2011 |
| EPI446997 | HA | Cambodia | 2011-Aug-17 | B/Cambodia/V0825316/2011 |
| EPI446996 | HA | Cambodia | 2011-Jun-24 | B/Cambodia/V0629331/2011 |
| EPI446992 | HA | Cambodia | 2011-May-30 | B/Cambodia/V0602318/2011 |
| EPI446991 | HA | Cambodia | 2011-Mar-10 | B/Cambodia/V0316324/2011 |
| EPI446990 | HA | Cambodia | 2011-Jan-06 | B/Cambodia/V0112324/2011 |
| EPI446989 | HA | Cambodia | 2011-Feb-01 | B/Cambodia/V0112308/2011 |
| EPI341534 | HA | Cambodia | 2009-Sep-08 | B/Cambodia/T0910051/2009 |
| EPI341532 | HA | Cambodia | 2009-Nov-10 | B/Cambodia/T1119143/2009 |
| EPI341530 | HA | Cambodia | 2009-Dec-29 | B/Cambodia/T1231151/2009 |
| EPI341528 | HA | Cambodia | 2010-Oct-10 | B/Cambodia/U1006332/2010 |
| EPI341526 | HA | Cambodia | 2010-Oct-06 | B/Cambodia/U1013320/2010 |
| EPI341525 | HA | Cambodia | 2010-Nov-22 | B/Cambodia/U1103348/2010 |
| EPI331319 | HA | Cambodia | 2011-Jan-04 | B/CAMBODIA/30/2011 |
| EPI370299 | HA | Cambodia | 2011-Dec-12 | B/CAMBODIA/106/2011 |
| EPI464421 | HA | Cambodia | 2005-Jul-10 | B/Cambodia/U0707330/2010 |
| EPI464422 | HA | Cambodia | 2010-Oct-26 | B/Cambodia/U1102389/2010 |
