# Supplementary material for: Epidemiological and Virological Characteristics of Influenza Viruses Circulating in Cambodia from 2009 to 2011
Source: PLoS One. 2014 Oct 23;9(10):e110713. doi: 10.1371/journal.pone.0110713 (PMC4207757; doi:10.1371/journal.pone.0110713)
Supplement: Table S3 — Resistance to adamantanes predicted following M gene sequencing of influenza A viruses, 2009–2011. (DOC) [file pone.0110713.s007.doc]

**Table S3.** **Resistance to adamantanes predicted following M gene sequencing of influenza A viruses, 2009 – 2011**.

|  | N# of predicted resistant isolates/total N# of isolates sequenced (%) | | |
| --- | --- | --- | --- |
| Year | H3N2 | H1N1pdm09 | H5N1 |
| 2009 | 14/14 (100) | 16/16 (100) | NA |
| 2010 | 15/15 (100) | 18/18 (100) | 1/1 (100) |
| 2011 | 5/5 (100) | 5/6 (83.3) | 7/7 (100) |
| Total | 34/34 (100) | 39/40 (97.5) | 8/8 (100) |

NA: no strains available.
