# Supplementary material for: Epidemiological and Virological Characteristics of Influenza Viruses Circulating in Cambodia from 2009 to 2011
Source: PLoS One. 2014 Oct 23;9(10):e110713. doi: 10.1371/journal.pone.0110713 (PMC4207757; doi:10.1371/journal.pone.0110713)
Supplement: Table S4 — Amino acid substitutions in A/H3N2 viruses isolated in Cambodia from 2009 to 2011. (DOCX) [file pone.0110713.s008.docx]

**Table S4. Amino acid substitutions in A/H3N2 viruses isolated in Cambodia from 2009 to 2011**

| **Residue position in HA1** | | | | | | | | | | | | | | | | | | | | | | | | | | | | | | | |
| --- | --- | --- | --- | --- | --- | --- | --- | --- | --- | --- | --- | --- | --- | --- | --- | --- | --- | --- | --- | --- | --- | --- | --- | --- | --- | --- | --- | --- | --- | --- | --- |
| **Strains** | **25** | **33** | **45** | **48** | **53** | **62** | **74** | **82** | **88** | **94** | **106** | **112** | **118** | **130** | **140** | **143** | **144** | **145** | **158** | **173** | **174** | **183** | **189** | **198** | **202** | **208** | **212** | **214** | **221** | **223** | **225** |
| **A/Brisbane/10/2007^a^** | **I** | **Q** | **S** | **T** | **D** | **E** | **P** | **K** | **V** | **Y** | **A** | **V** | **L** | **V** | **I** | **S** | **N** | **N** | **K** | **K** | **F** | **H** | **N** | **A** | **I** | **R** | **T** | **I** | **P** | **V** | **N** |
| A/Cambodia/T092/2009 | V | - | - | - | - | K | - | - | - | - | - | - | - | - | - | - | K | - | N | Q | - | - | K | - | - | - | - | - | - | - | - |
| A/Cambodia/T085/2009 | V | - | - | - | - | K | - | - | - | - | - | - | - | L | - | - | K | - | N | Q | S | - | K | - | - | - | - | - | - | - | - |
| A/Cambodia/T103/2009 | - | - | - | - | - | K | - | - | - | - | - | - | - | - | - | - | K | - | N | Q | - | - | K | - | - | - | - | - | - | - | - |
| A/Cambodia/T245/2009 | - | - | - | - | - | K | - | N | - | H | - | - | M | - | - | - | K | - | N | Q | - | - | K | - | - | - | - | - | - | - | - |
| A/Cambodia/7/2009 | - | - | - | - | - | K | - | - | - | H | - | - | - | - | - | - | K | - | N | Q | - | - | K | - | - | - | - | - | - | I | - |
| A/Cambodia/T140/2009 | - | - | - | - | - | K | - | - | - | H | - | - | - | - | - | - | K | - | N | Q | - | - | K | - | T | - | - | - | - | I | - |
| A/Cambodia/T308/2009 | - | - | - | - | - | K | - | T | - | H | - | - | - | - | - | Y | K | - | N | Q | - | - | K | - | - | - | - | - | - | - | - |
| A/Cambodia/15/2009 | - | - | - | - | - | K | - | - | - | H | - | - | - | - | - | - | K | - | N | Q | - | - | K | - | - | - | - | - | - | - | - |
| A/Cambodia/T284/2009 | - | - | - | - | - | K | - | - | - | H | - | - | - | - | - | - | K | - | N | Q | - | - | K | - | - | - | - | - | - | - | - |
| A/Cambodia/T108/2009 | - | - | - | - | - | K | - | - | - | H | - | - | - | - | - | - | K | - | N | Q | - | - | K | - | - | - | - | - | - | - | - |
| A/Cambodia/12/2009 | - | - | - | - | - | K | - | - | - | H | - | - | - | - | - | - | K | - | N | Q | - | - | K | - | - | - | - | - | - | - | - |
| A/Cambodia/U0825342/2010 | - | - | - | - | - | K | - | - | - | H | - | - | - | - | - | - | K | - | N | Q | - | - | K | - | - | - | - | - | - | - | - |
| **A/Perth/16/2009^b^** | - | - | - | - | - | K | - | - | - | - | - | - | - | - | - | - | K | - | N | Q | - | L | K | - | - | - | - | S | - | - | - |
| A/Cambodia/40/2011 | - | - | N | I | - | - | - | - | - | - | - | I | - | - | M | - | - | S | N | Q | - | - | K | S | - | - | A | - | - | I | - |
| A/Cambodia/V1116321/2011 | - | - | N | I | - | - | - | - | - | - | - | - | - | - | L | - | S | C | N | Q | - | - | K | S | - | - | A | - | - | I | - |
| A/Cambodia/V1013305/2011 | - | - | N | I | - | - | - | - | - | - | - | - | - | - | M | - | - | S | N | Q | - | - | K | S | - | - | A | - | - | I | - |
| A/Cambodia/55/2011 | - | - | N | I | - | - | - | - | - | - | - | - | - | - | M | - | - | S | N | Q | - | - | K | S | - | - | A | - | - | I | - |
| A/Cambodia/74/2011 | - | R | N | I | - | - | - | - | - | - | - | - | - | - | - | - | - | - | N | Q | - | - | K | S | - | - | A | - | T | I | - |
| A/Cambodia/V1116330/2011 | - | R | N | I | - | - | L | - | - | - | T | - | - | - | - | - | - | - | N | Q | - | - | K | S | - | - | A | - | L | I | - |
| A/Cambodia/V0902310/2011 | - | R | N | I | - | - | - | - | I | - | - | - | - | - | - | - | - | - | N | Q | - | - | K | S | - | - | A | - | - | I | D |
| A/Cambodia/V1005380/2011 | - | R | N | I | - | - | - | - | - | - | - | - | - | - | - | - | - | - | N | Q | - | - | K | S | - | - | A | - | - | I | - |
| A/Cambodia/39/2011 | - | R | N | I | - | - | - | - | - | - | - | - | - | - | - | - | - | - | N | Q | - | - | K | S | - | - | A | - | - | I | - |
| A/Cambodia/U307/2010 | - | - | - | - | - | - | - | - | - | H | - | - | - | - | - | - | - | - | N | Q | - | - | K | - | - | - | A | - | - | - | - |
| A/Cambodia/U371/2010 | - | - | - | - | N | - | - | - | - | H | - | - | - | - | - | - | - | - | N | Q | - | - | K | - | - | K | A | - | - | - | - |
| A/Cambodia/U368/2010 | - | - | - | - | N | - | - | - | - | H | - | - | - | - | - | - | - | - | N | Q | - | - | K | - | - | K | A | - | - | - | - |
| A/Cambodia/U5340/2010 | - | - | - | - | N | - | - | - | - | H | - | - | - | - | - | - | - | - | N | Q | - | - | K | - | - | K | A | - | - | - | - |
| A/Cambodia/U424/2010 | - | - | - | - | N | - | - | - | - | H | - | - | - | - | - | - | - | - | N | Q | - | - | K | - | - | - | A | - | - | - | - |
| A/Cambodia/U325/2010 | - | - | - | - | N | - | - | - | - | H | - | - | - | - | - | - | - | - | N | Q | - | - | K | - | - | - | A | - | - | - | - |
| A/Cambodia/U349/2010 | - | - | - | - | N | - | - | - | - | H | - | - | - | - | - | - | - | - | N | Q | - | - | K | - | - | - | A | - | L | - | - |

| **Residue position in HA1** | | | | | | | | | |
| --- | --- | --- | --- | --- | --- | --- | --- | --- | --- |
| **Strains** | **230** | **242** | **278** | **280** | **287** | **289** | **309** | **312** | **329** |
| **A/Brisbane/10/2007^a^** | **I** | **I** | **N** | **E** | **S** | **P** | **V** | **N** | **R** |
| A/Cambodia/T092/2009 | - | - | - | - | - | - | - | - | - |
| A/Cambodia/T085/2009 | - | - | - | - | - | - | - | - | - |
| A/Cambodia/T103/2009 | - | - | - | - | - | - | - | - | - |
| A/Cambodia/T245/2009 | - | - | - | - | - | - | - | - | - |
| A/Cambodia/7/2009 | - | - | - | - | - | - | - | - | - |
| A/Cambodia/T140/2009 | - | L | - | - | - | - | - | - | K |
| A/Cambodia/T308/2009 | - | L | - | - | - | - | - | - | K |
| A/Cambodia/15/2009 | - | L | - | - | - | - | - | - | K |
| A/Cambodia/T284/2009 | - | L | - | - | - | - | - | - | K |
| A/Cambodia/T108/2009 | - | L | - | - | - | - | - | - | K |
| A/Cambodia/12/2009 | - | L | - | - | - | - | - | - | K |
| A/Cambodia/ U0825342/2010 | - | L | - | - | - | - | - | - | K |
| **A/Perth/16/2009^b^** | - | - | - | - | - | - | - | - | - |
| A/Cambodia/40/2011 | - | - | - | - | - | - | I | S | - |
| A/Cambodia/V1116321/2011 | - | - | - | - | - | - | - | S | - |
| A/Cambodia/V1013305/2011 | - | - | - | - | - | - | - | S | - |
| A/Cambodia/55/2011 | - | - | - | - | - | - | - | S | - |
| A/Cambodia/74/2011 | - | - | K | - | - | - | - | S | - |
| A/Cambodia/V1116330/2011 | - | - | K | - | - | - | - | S | - |
| A/Cambodia/V0902310/2011 | - | - | K | - | - | - | - | S | - |
| A/Cambodia/V1005380/2011 | - | - | K | - | - | - | - | S | - |
| A/Cambodia/39/2011 | - | - | K | - | - | - | - | S | - |
| A/Cambodia/U307/2010 | V | - | - | A | - | T | - | - | - |
| A/Cambodia/U371/2010 | V | - | - | A | - | - | - | - | - |
| A/Cambodia/U368/2010 | V | - | - | A | - | - | - | - | - |
| A/Cambodia/U5340/2010 | V | - | - | A | - | - | - | - | - |
| A/Cambodia/U424/2010 | V | - | - | A | C | - | - | - | - |
| A/Cambodia/U325/2010 | V | - | - | A | - | - | - | - | - |
| A/Cambodia/U349/2010 | V | - | - | A | - | - | - | - | - |

Identity to reference vaccine strain A/Brisbane/10/2007 is indicated by a dash. ^a^ vaccine strain in 2009-2010, ^b^ vaccine strain in 2010-2011.
