# Supplementary material for: Epidemiological and Virological Characteristics of Influenza Viruses Circulating in Cambodia from 2009 to 2011
Source: PLoS One. 2014 Oct 23;9(10):e110713. doi: 10.1371/journal.pone.0110713 (PMC4207757; doi:10.1371/journal.pone.0110713)
Supplement: Table S5 — Amino acid substitutions in A/H1N1pdm09 viruses isolated in Cambodia from 2009 to 2011. (DOCX) [file pone.0110713.s009.docx]

**Table S5. Amino acid substitutions in A/H1N1pdm09 viruses isolated in Cambodia from 2009 to 2011.**

| **Residue position in HA1** | | | | | | | | | | | | | | | | | | | | | | | | | | | | | | | | |
| --- | --- | --- | --- | --- | --- | --- | --- | --- | --- | --- | --- | --- | --- | --- | --- | --- | --- | --- | --- | --- | --- | --- | --- | --- | --- | --- | --- | --- | --- | --- | --- | --- |
| **Strains** | **5** | **19** | **37** | **70** | **71** | **83** | **84** | **86** | **97** | **106** | **116** | **121** | **125** | **143** | **149** | **156** | **160** | **171** | **183** | **184** | **185** | **197** | **202** | **203** | **205** | **215** | **223** | **227** | **260** | **293** | **321** | **327** |
| **A/California/07/2009^a^** | **I** | **V** | **H** | **L** | **S** | **P** | **S** | **D** | **D** | **S** | **I** | **S** | **N** | **S** | **I** | **N** | **K** | **K** | **S** | **T** | **S** | **A** | **G** | **S** | **R** | **A** | **R** | **M** | **N** | **Q** | **I** | **R** |
| A/Cambodia/T021/2009 | - | - | - | - | - | S | - | - | - | - | - | - | - | - | - | - | - | - | - | - | - | - | - | - | - | - | Q | - | - | H | V | - |
| A/Cambodia/T320/2009 | - | - | - | - | - | S | - | - | - | - | - | - | - | - | - | - | - | - | - | - | - | - | - | T | - | - | Q | - | - | - | V | - |
| A/Cambodia/T354/2009 | - | - | - | - | - | S | - | - | - | - | - | - | - | - | - | - | - | - | - | - | - | - | - | T | - | - | Q | - | - | - | V | - |
| A/Cambodia/T093/2009 | - | - | - | - | - | S | - | G | - | - | - | - | - | - | - | - | - | - | - | - | - | - | - | T | - | - | Q | - | - | - | V | - |
| A/Cambodia/U094/2010 | - | - | N | - | - | S | - | - | - | - | M | - | - | - | - | - | - | - | - | - | - | - | - | T | - | - | Q | - | - | - | V | - |
| A/Cambodia/T217/2009 | - | - | - | - | - | S | - | - | - | - | - | - | - | - | - | - | - | - | - | - | - | - | - | T | - | - | Q | - | D | - | V | - |
| A/Cambodia/T282/2009 | - | - | - | - | - | S | - | - | - | - | - | - | - | - | - | - | - | - | - | - | - | - | - | T | K | - | Q | - | - | - | V | - |
| A/Cambodia/T272/2009 | - | - | - | - | - | S | - | - | - | - | - | - | - | - | - | - | - | - | - | - | - | - | - | T | - | - | Q | - | - | - | V | - |
| A/Cambodia/T057/2009 | - | - | - | - | - | S | - | - | - | - | - | - | - | - | - | - | - | - | - | - | - | - | - | T | K | - | Q | - | - | - | V | - |
| A/Cambodia/U301/2010 | - | - | - | - | A | S | N | - | - | - | - | - | - | - | - | - | - | R | - | - | - | T | - | T | - | - | Q | - | - | - | V | - |
| A/Cambodia/T234/2009 | - | - | - | - | - | S | - | - | - | - | - | - | - | - | - | - | - | - | - | - | - | - | - | T | - | - | Q | - | - | - | V | - |
| A/Cambodia/T068/2009 | - | - | - | - | - | S | - | - | - | - | - | - | - | - | - | - | - | - | - | - | - | - | - | T | - | - | Q | - | - | - | V | - |
| A/Cambodia/T075/2009 | - | - | - | - | - | S | - | - | - | - | - | - | - | - | - | - | - | - | - | - | - | - | - | T | - | - | Q | - | - | - | V | - |
| A/Cambodia/T028/2009 | V | - | - | - | - | S | - | - | - | - | - | - | - | - | - | - | - | - | - | - | - | - | - | T | - | - | Q | - | - | - | V | - |
| A/Cambodia/U219/2010 | - | - | - | - | - | S | - | - | - | - | - | - | - | - | - | - | - | - | - | - | - | - | - | T | - | - | Q | - | - | - | V | - |
| A/Cambodia/U306/2010 | - | - | - | - | - | S | - | - | - | - | - | - | - | - | - | - | - | - | - | - | - | - | - | T | - | - | Q | - | - | - | V | - |
| A/Cambodia/U326/2010 | - | - | - | - | - | S | - | - | - | - | - | - | D | - | - | - | - | - | - | - | - | - | - | T | - | - | Q | - | - | - | V | - |
| A/Cambodia/U099/2010 | - | - | - | - | - | S | - | - | - | - | - | - | D | - | - | - | - | - | - | - | - | - | - | T | - | - | Q | - | - | - | V | - |
| A/Cambodia/9/2010 | - | - | - | - | - | S | - | - | - | - | - | - | - | - | - | - | - | - | - | - | - | - | - | T | - | - | Q | - | - | - | V | - |
| A/Cambodia/8/2010 | - | - | - | - | - | S | - | - | - | - | - | - | - | - | - | - | - | - | - | - | - | - | - | T | - | - | Q | - | - | - | V | - |
| A/Cambodia/10/2010 | - | - | - | - | - | S | - | - | - | - | - | - | - | - | - | - | - | - | - | - | - | - | - | T | - | - | Q | - | - | - | V | - |
| A/Cambodia/72/2010 | - | - | - | - | - | S | - | - | - | - | - | - | - | - | - | - | - | - | - | - | T | T | - | T | - | - | Q | - | - | - | V | - |
| A/Cambodia/V0608350/2011 | - | - | - | - | - | S | - | - | - | - | - | - | D | G | - | - | - | - | - | - | T | T | - | T | - | - | Q | - | - | - | V | - |
| A/Cambodia/V0721310/2011 | - | - | - | - | - | S | - | - | - | - | - | - | - | G | - | D | - | - | - | N | T | T | - | T | - | - | Q | - | G | - | V | K |
| A/Cambodia/15/2011 | - | - | - | - | - | S | - | - | - | - | - | - | - | G | - | - | - | - | - | - | I | - | - | T | - | - | Q | V | D | - | V | - |
| A/Cambodia/69/2011 | - | - | - | - | - | S | - | - | - | - | - | - | - | G | - | - | T | - | - | - | T | T | - | T | - | - | Q | - | D | - | V | - |
| A/Cambodia/70/2011 | - | I | - | - | - | S | - | - | - | - | - | - | - | G | - | - | - | - | - | - | T | T | - | T | - | - | Q | - | D | - | V | - |
| A/Cambodia/V0601312/2011 | - | - | - | - | - | S | - | - | - | - | - | - | - | G | - | - | - | - | - | - | T | T | W | T | - | - | Q | - | D | - | V | - |
| A/Cambodia/V0902314/2011 | - | - | - | F | - | S | - | - | - | - | - | - | - | G | V | - | - | - | P | - | T | T | - | T | - | - | Q | - | D | - | V | - |
| A/Cambodia/V1005346/2011 | - | - | - | - | - | S | - | - | - | - | - | - | - | G | - | - | - | - | - | - | T | T | - | T | - | T | Q | - | D | - | V | - |
| A/Cambodia/V1019320/2011 | - | - | - | - | - | S | - | - | - | N | - | - | - | G | - | - | - | - | - | - | T | T | E | T | - | - | Q | - | D | H | V | - |
| A/Cambodia/59/2011 | - | - | - | - | - | S | - | - | - | - | - | - | - | G | - | - | - | - | - | - | T | T | - | T | - | - | Q | - | D | - | V | - |
| A/Cambodia/53/2011 | - | - | - | - | - | S | - | - | N | - | - | N | - | G | - | - | - | - | - | - | T | T | - | T | - | - | Q | - | D | - | V | - |

| **Residue position in HA2** | | | | | | | | | | | | |
| --- | --- | --- | --- | --- | --- | --- | --- | --- | --- | --- | --- | --- |
| **Strains** | **374** | **411** | **423** | **442** | **451** | **454** | **460** | **474** | **476** | **477** | **499** | **501** |
| **A/California/07/2009^a^** | **E** | **V** | **A** | **V** | **S** | **K** | **I** | **T** | **M** | **E** | **E** | **D** |
| A/Cambodia/T021/2009 | - | - | T | - | - | - | - | - | - | - | - | - |
| A/Cambodia/T320/2009 | - | - | - | - | - | - | - | - | - | - | - | - |
| A/Cambodia/T354/2009 | - | - | - | - | - | - | - | - | - | - | - | - |
| A/Cambodia/T093/2009 | - | - | - | - | - | - | - | - | - | - | - | - |
| A/Cambodia/U094/2010 | - | - | - | - | - | - | V | - | - | - | - | - |
| A/Cambodia/T217/2009 | - | I | - | - | - | - | - | - | - | - | - | - |
| A/Cambodia/T282/2009 | - | I | - | - | - | - | - | - | - | - | - | - |
| A/Cambodia/T272/2009 | - | I | - | - | - | - | - | - | - | - | - | - |
| A/Cambodia/T057/2009 | - | - | - | - | - | - | - | - | - | - | - | - |
| A/Cambodia/U301/2010 | - | - | - | - | - | - | - | - | - | - | - | - |
| A/Cambodia/T234/2009 | - | - | - | - | - | - | - | - | - | - | - | - |
| A/Cambodia/T068/2009 | - | - | - | - | - | - | - | - | - | - | - | - |
| A/Cambodia/T075/2009 | - | - | - | - | - | - | - | - | - | - | - | - |
| A/Cambodia/T028/2009 | - | - | - | - | - | - | - | - | - | - | - | - |
| A/Cambodia/U219/2010 | K | - | - | - | - | - | - | - | - | - | - | - |
| A/Cambodia/U306/2010 | K | - | - | - | - | - | - | - | - | - | - | N |
| A/Cambodia/U326/2010 | K | - | - | - | - | - | - | - | - | - | - | - |
| A/Cambodia/U099/2010 | K | - | - | - | - | - | - | - | - | - | - | - |
| A/Cambodia/9/2010 | K | - | - | - | N | - | - | - | - | - | - | - |
| A/Cambodia/8/2010 | K | - | - | - | - | - | - | - | - | - | - | - |
| A/Cambodia/10/2010 | K | - | - | - | - | - | - | - | - | - | - | - |
| A/Cambodia/72/2010 | K | - | - | L | N | - | - | - | - | - | - | - |
| A/Cambodia/V0608350/2011 | K | - | - | - | N | - | - | - | - | - | - | - |
| A/Cambodia/V0721310/2011 | K | - | - | - | N | - | - | - | - | - | K | - |
| A/Cambodia/15/2011 | K | - | - | - | N | - | - | - | - | - | K | - |
| A/Cambodia/69/2011 | K | - | - | - | N | - | - | - | T | - | K | - |
| A/Cambodia/70/2011 | K | - | - | - | N | - | - | K | - | - | K | - |
| A/Cambodia/V0601312/2011 | K | - | - | - | N | - | - | - | - | - | K | - |
| A/Cambodia/V0902314/2011 | K | - | - | - | N | E | - | - | - | K | K | - |
| A/Cambodia/V1005346/2011 | K | - | - | - | N | - | - | - | I | - | K | - |
| A/Cambodia/V1019320/2011 | K | - | - | - | N | - | - | - | - | - | K | - |
| A/Cambodia/59/2011 | K | - | - | - | N | - | - | - | - | - | K | - |
| A/Cambodia/53/2011 | K | - | - | - | N | - | - | - | - | - | K | - |

Identity to reference vaccine strain A/California/07/2009 is indicated by a dash. ^a^ Current vaccine strain.
