# Supplementary material for: Epidemiological and Virological Characteristics of Influenza Viruses Circulating in Cambodia from 2009 to 2011
Source: PLoS One. 2014 Oct 23;9(10):e110713. doi: 10.1371/journal.pone.0110713 (PMC4207757; doi:10.1371/journal.pone.0110713)
Supplement: Table S6 — Variations in HA1 amino acid sequences in influenza B-Victoria lineage viruses isolated in Cambodia in 2009, 2010 and 2011 by comparison with vaccine strain B/Malaysia/2506/2004. (DOCX) [file pone.0110713.s010.docx]

**Supplementary Table 6. Variations in HA1 amino acid sequences in influenza B-Victoria lineage viruses isolated in Cambodia in 2009, 2010 and 2011 by comparison with vaccine strain B/Malaysia/2506/2004.**

| **Virus Strain(s)** | **Residue position in HA1** | | | | | | | | | | | | | | | | | |
| --- | --- | --- | --- | --- | --- | --- | --- | --- | --- | --- | --- | --- | --- | --- | --- | --- | --- | --- |
|  | **7** | **31** | **37** | **56** | **58** | **75** | **90** | **129** | **134** | **146** | **154** | **162** | **165** | **172** | **197** | **199** | **255** | **345** |
| **B/Malaysia/2506/2004** | **I** | **P** | **T** | **K** | **L** | **N** | **V** | **N** | **S** | **V** | **A** | **K** | **N** | **S** | **N** | **A** | **S** | **K** |
| B/Cambodia/V0112324/2011 | - | - | I | - | - | - | - | - | P | - | - | - | - | - | - | T | - | - |
| B/Cambodia/30/2011 | - | S | I | - | - | - | - | - | P | - | - | - | - | - | S | T | - | - |
| B/Cambodia/V0316324/2011 | - | - | I | - | - | - | I | - | P | - | - | - | - | - | - | T | - | - |
| B/Cambodia/U1103348/2010 | - | - | I | - | - | - | - | - | P | - | - | - | - | - | - | T | - | - |
| B/Cambodia/V0629331/2011 | - | - | I | - | - | - | - | - | P | - | - | - | - | - | - | T | - | - |
| B/Cambodia/V0602318/2011 | - | - | I | - | - | - | - | - | P | - | - | - | - | - | - | T | - | - |
| **B/Brisbane/60/2008**^*^ | - | - | - | - | - | K | - | - | P | I | - | - | K | P | - | T | - | - |
| B/Cambodia/T1231151/2009 | V | - | - | R | - | K | - | - | P | I | - | - | K | P | - | T | - | - |
| B/Cambodia/T0910051/2009 | - | - | - | R | - | K | - | - | P | I | - | - | K | P | - | T | - | - |
| B/Cambodia/T1119143/2009 | - | - | - | R | - | K | - | - | P | I | - | - | K | P | - | T | - | - |
| B/Cambodia/U1102389/2010 | - | - | - | R | - | K | - | - | P | I | - | - | K | P | - | T | - | R |
| B/Cambodia/U0707330/2010 | - | - | - | R | - | K | - | - | P | I | - | N | K | P | - | T | - | - |
| B/Cambodia/V1221355/2011 | - | - | - | - | - | K | - | D | P | - | - | - | K | P | - | T | - | - |
| B/Cambodia/106/2011 | - | - | - | - | - | K | - | D | P | - | - | - | K | P | - | T | - | - |
| B/Cambodia/V0825326/2011 | - | - | - | - | - | K | - | D | P | - | - | - | K | P | - | T | - | - |
| B/Cambodia/V1103320/2011 | - | - | - | - | - | K | - | - | P | - | E | - | K | P | - | T | P | - |
| B/Cambodia/V1012330/2011 | - | - | - | - | - | K | - | - | P | - | E | - | K | P | - | T | - | - |
| B/Cambodia/V0825316/2011 | - | - | - | - | - | K | - | - | P | - | E | - | K | P | - | T | - | - |
| B/Cambodia/V0112308/2011 | - | - | - | - | P | K | - | - | P | - | - | - | K | P | - | T | - | - |
| B/Cambodia/U1013320/2010 | - | - | - | - | P | K | - | - | P | - | - | - | K | P | - | T | - | - |
| B/Cambodia/U1006332/2010 | - | - | - | - | P | K | - | - | P | - | - | - | K | P | - | T | - | - |

Identity to reference vaccine strain B/Malaysia/2506/2004 is indicated by a dash. ^*^ Current vaccine strain.
